# Supplementary material for: Diversity and evolution of a phase-variable multi-locus antigen in Neisseria gonorrhoeae
Source: PLoS Pathog. 2026 May 11;22(5):e1013962. doi: 10.1371/journal.ppat.1013962 (PMC13183285; doi:10.1371/journal.ppat.1013962)
Supplement: S1 File — (DOCX) [file ppat.1013962.s001.docx]

**Supplementary Information**

Supplementary Methods

*Short-read sequencing*

Short-read sequencing for EEE029 was performed in Bristow, Mortimer, et al. [75] and short-read sequencing of UMASS-DGI_65 was performed using the same method. Briefly, DNA was extracted from bacterial cells grown overnight on GCB-K plates at 37°C with 5% carbon dioxide using the Invitrogen PureLink Genomic DNA Mini Kit. Libraries were prepped and sequenced on the Illumina NextSeq 2000 sequencer at the Microbial Whole Genome Sequencing Center or the Bauer Core Facility at Harvard University.

*Comparison of long-read assembly and polishing methods*

To develop a pipeline for assembling complete genomes to minimize errors in the *opa* genes, we used the following assembly and polishing approaches using the filtered long-reads from Oxford Nanopore (see Methods) and short reads generated by Illumina NextSeq: Flye v2.9.5 [31], Medaka v2.0.1 (https://github.com/nanoporetech/medaka), Polypolish v0.6.0 [76], Pypolca v0.3.1 [76], and Autocycler v0.2.1 [29]. We tested the following workflows:

1. Long-read only assembly with Flye.
2. Long-read only assembly with Flye 🡪 long-read polishing with Medaka
3. Long-read only assembly with Flye 🡪 long-read polishing with Medaka 🡪 short-read polishing with Polypolish default
4. Long-read only assembly with Flye 🡪 long-read polishing with Medaka 🡪 short-read polishing with Polypolish default followed by short-read polishing with Pypolca careful
5. Long-read only assembly with Autocycler
6. Long-read only assembly with Autocycler 🡪 short-read polishing with Polypolish default
7. Long-read only assembly with Autocycler 🡪 short-read polishing with Polypolish default 🡪 short-read polishing with Pypolca careful

We used the genomes UMASS-DGI_65 and EEE029 which had sequencing depth of around 140x. To test how sequencing depth would affect the *opa* sequence accuracy, we randomly subsampled the reads using the seqtk package v1.4 (<https://github.com/lh3/seqtk>) to 15% (21x), 30% (42x), and 60% (84x), and compared to the results including 100% of the reads (140x). For each read subset, we assembled the genome using the approaches above and identified the *opa* sequences as described in the Methods. We aligned the resulting *opa* sequences using MAFFT v7.520 [39] with the default parameters.

*More extensive testing of Autocycler with different read depth across phylogenetically diverse isolates*

We selected 11 phylogenetically diverse isolates with at least 125x coverage using Treemmer v0.3 [77]. We created 10 random read subsets using the seqtk package at 50x, 75x, 100x, and 125x coverage. We assembled the genomes using the following approaches. We assembled the genomes (using long reads only) using Autocycler and followed the approach above to compare *opa* sequences.

*Algorithm to search for* opa *genes in the complete genomes*

We wrote a custom python script to identify *opa* genes (**S1 Fig**). First, we searched for 3 to 100 tandem repeats of the CTCTT pentanucleotide with at most 2 substitutions across the entire sequence, which we will refer to as the coding repeats (CR), similar to the approach used in Bilek et al. [24]. We then searched for the unique conserved sequence near the stop codon (TGCGCTACCGCTTCTGAT) with at most 2 substitutions, which we will refer to as the term sequence. Pairs of CR and term sequences were matched if they were separated by less than 1200 bp in the genome. For term sequences that were unpaired, a more lenient search was performed for the upstream CR where we allowed up to 1 mismatch for each CR unit (substitution, insertion, or deletion) to catch CR units that contained mutations. If there was still not a matching CR found, it could have been that the *opa* did not have a CR; in this case, we looked for the sequence found directly upstream of the CR consisting of a poly-A sequence of length 5-7 followed by CCTT and allowed one mismatch (substitution, insertion, or deletion).

The start codon (ATG) was identified in the 50 bp upstream of the start of the CR sequence and the stop codon (TGA) was contained in the term sequence. Occasionally, the beginning and end of the CR region was not defined precisely with the above procedure due to mimatches in the CR (substitution, insertion, or deletion). Thus, the start of the CR was made more precise by finding the upstream sequence (see end of previous paragraph). If this sequence was not found, then the start of CR from earlier was used. Similarly, the end of the CR was made more precise by searching for the downstream sequence, which is CCG, allowing for the first C to be optional and 1 additional mismatch.

*Calculation of the randomness of hypervariable 1 and hypervariable 2 allele types*

We dropped duplicate *opa* genes in the same genome as determined by having the same semivariable, hypervariable 1, and hypervariable 2 cluster type combinations to remove the effect of recent gene conversion events that have not yet had the time to be subject to selection.

We accounted for isolate sampling and *N. gonorrhoeae* population structure by partitioning the recombination-corrected phylogeny of complete genomes using fastbaps v1.0.8 [78] with the BAPS prior. We randomly selected 1 isolate from each BAPS cluster.

We determined the number of times each hypervariable 1 and hypervariable 2 cluster types appeared together in all *opa* genes in the subsampled isolates and created an association matrix where the rows were hypervariable 1 types, the columns were hypervariable 2 types, and the element values were the number of times each combination appeared. Because the number of hypervariable 1 and hypervariable 2 types were unequal, we padded columns with zeros to give a square matrix. We determined the degree of association by rearranging the columns of the association matrix to maximize the sum of diagonal entries using the Munkres algorithm (<https://github.com/bmc/munkres?tab=readme-ov-file>, v1.1.4). To determine whether the observed data could be random, we randomized the hypervariable 2 cluster types across the *opa* genes 100 times. We compared the maximum sum of diagonal elements from the observed data and the randomized data. We then repeated this process by performing 99 random subsamples of the isolates in each BAPS cluster.

*Quantification of frameshift mutations downstream of the coding repeats leading to a premature stop codon*

We identified all *opa* amino acid sequences that had a premature stop codon after the end of the coding repeat sequence. The approximate genome coverage was calculated as the number of input read bases divided by the number of consensus assembly bases. To identify the location of the frameshift mutations, the nucleotide sequences were aligned with MAFFT v7.520 [39] with the default parameters and visualized in Jalview v2.11.4.1 [40].

Comparison of long-read assembly and polishing methods

We first compared multiple assembly and polishing approaches, including Flye, Medaka, Polypolish, Pypolca, and Autocycler at 4 read depths (21x, 42x, 84x, and 140x) for two genomically diverse isolates. The *opa* sequences were identical across assemblies except for 1 SNP in UMASS-DGI_65 *opa7* and a 1 base insertion in EEE029 *opa11*, both of which occurred in methods 1 (long-read assembly with Flye) and 2 (long-read assembly with Flye followed by long-read polishing with Medaka) for a read depth of 21x (**S3 Fig**). This analysis suggested Autocycler was the best performing assembly method and that polishing the Autocycler assemblies with short reads did not affect the *opa* sequences. However, because we only tested 2 genomes, we wanted to expand the analysis to include more isolates that are representative of *N. gonorrhoeae* diversity and to subset the reads to test lower read depths more systemically.

In our more extensive testing of Autocycler using 11 phylogenetically diverse isolates and 4 read depths (50x, 75x, 100x, and 125x) the *opa* sequences were identical across assemblies except for the following differences (always compared to 125x coverage assembly) (**S4 Fig**):

- EEE023 *opa4* had 1 SNP in 10/10 assemblies with 50x coverage.
- FFF007 *opa11* was not detected in 10/10 assemblies with 50x coverage due to a missing stop codon and had multiple sequence differences in 10/10 assemblies with 75x coverage.
- GCGS0313 *opa1* had a 1 base deletion in 9/10 assemblies with 125x coverage
- GCGS0313 *opa5* had 1 SNP in 10/10 assemblies with 50x coverage

Despite these changes in a small number of *opa* sequences, we concluded that most *opa* sequences were identical across read depths and that Autocycler assemblies were good enough for our purposes of looking at diversity and evolution across a large set of *opa* genes.

**References**

75. Bristow CC, Mortimer TD, Morris S, Grad YH, Soge OO, Wakatake E, et al. Whole-genome sequencing to predict antimicrobial susceptibility profiles in Neisseria gonorrhoeae. J Infect Dis. 2023;227: 917–925.

76. Bouras G, Judd LM, Edwards RA, Vreugde S, Stinear TP, Wick RR. How low can you go? Short-read polishing of Oxford Nanopore bacterial genome assemblies. Microbial Genomics. 2024;10:001254.

77. Menardo F, Loiseau C, Brites D, Coscolla M, Gygli SM, Rutaihwa LK, et al. Treemmer: a tool to reduce large phylogenetic datasets with minimal loss of diversity. BMC Bioinformatics. 2018;19: 164.

78. Tonkin-Hill G, Lees JA, Bentley SD, Frost SDW, Corander J. Fast hierarchical Bayesian analysis of population structure. Nucleic Acids Res. 2019;47: 5539–5549.
