# Supplementary figures and images for: Diversity and evolution of a phase-variable multi-locus antigen in Neisseria gonorrhoeae
Source: PLoS Pathog. 2026 May 11;22(5):e1013962. doi: 10.1371/journal.ppat.1013962 (PMC13183285; doi:10.1371/journal.ppat.1013962)

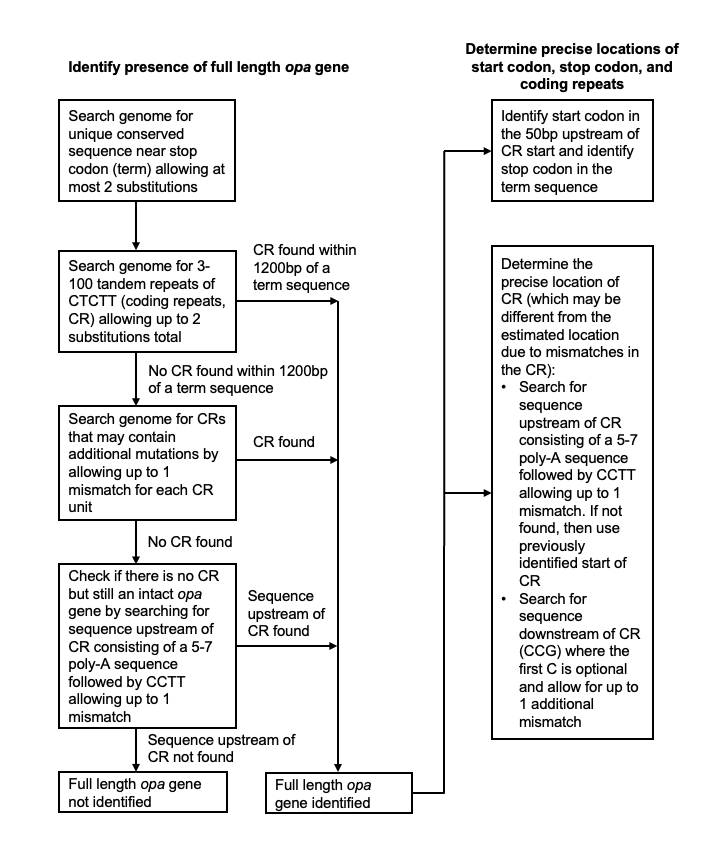

Supplement: S1 Fig — Note that a mismatch refers to a substitution, insertion, or deletion. (PNG) [file ppat.1013962.s002.png]

Continent

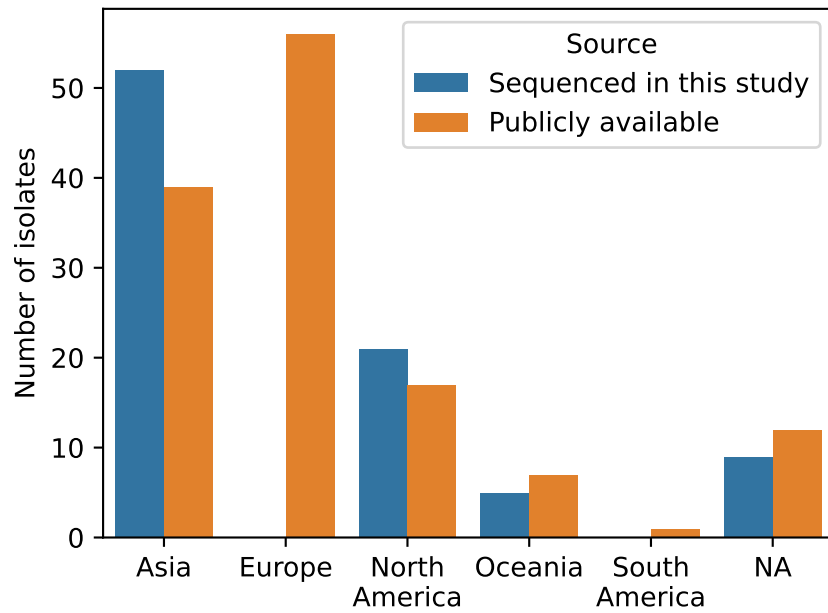

Gender

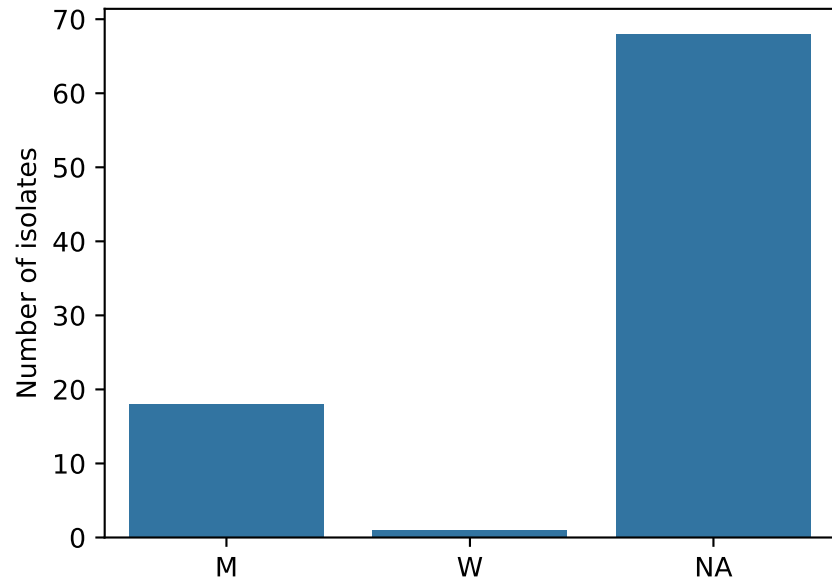

Year of isolation

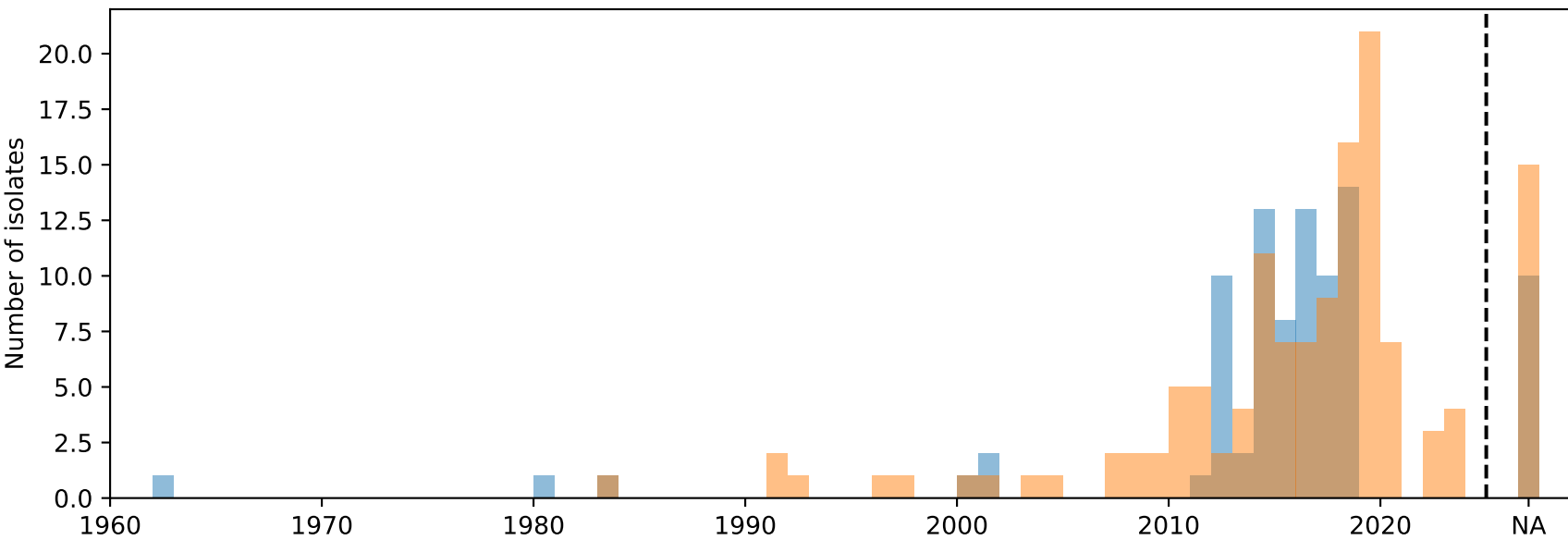

Supplement: S2 Fig — The information on host gender was not included with NCBI metadata for the publicly available complete genomes. Abbreviations: Man (M), Woman (W), Not available (NA). (PDF) [file ppat.1013962.s003.pdf]

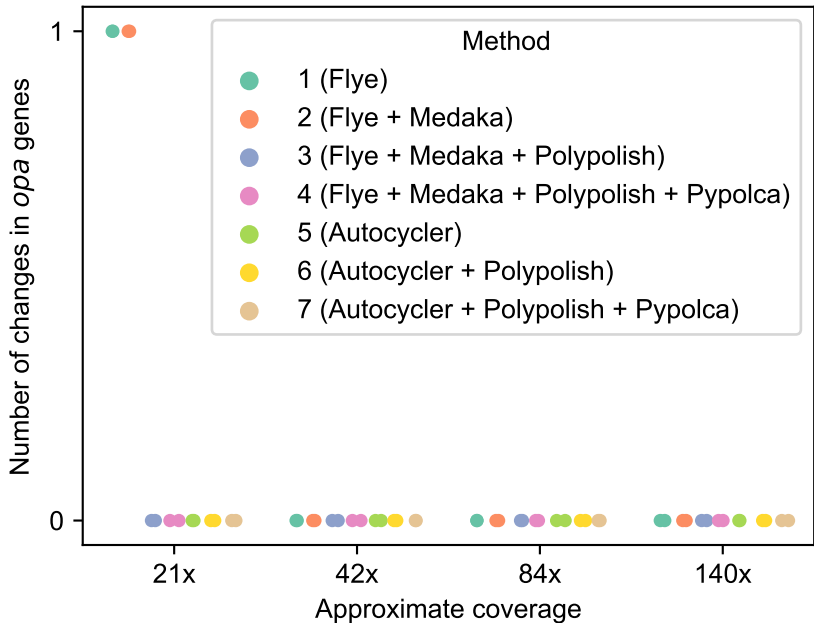

Supplement: S3 Fig — The number of changes in opa genes at 4 read coverage levels using 7 different assembly and polishing procedures. There are two points of the same color in each coverage level, indicating two different isolates. The changes at 21x for methods 1 and 2 include one SNP in one isolate’s genome and one single base insertion in the other isolate’s genome. (PDF) [file ppat.1013962.s004.pdf]

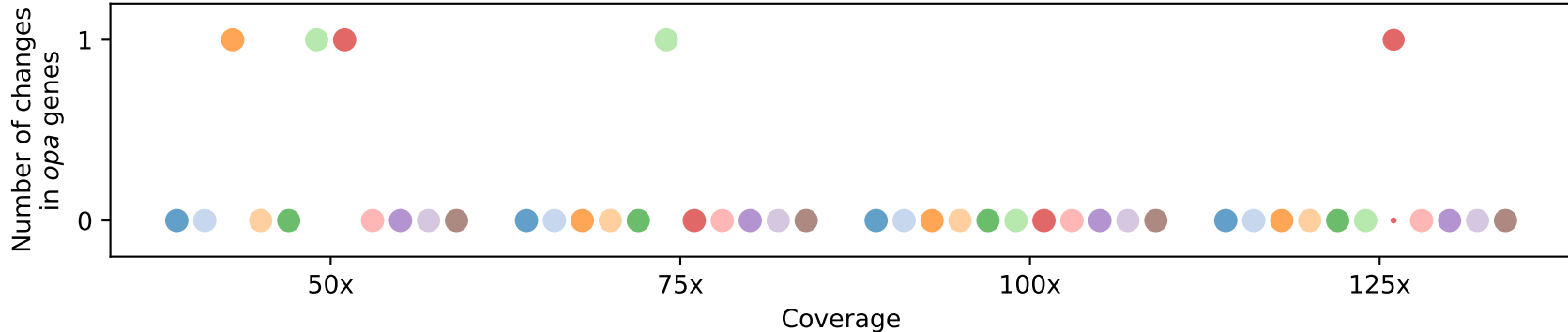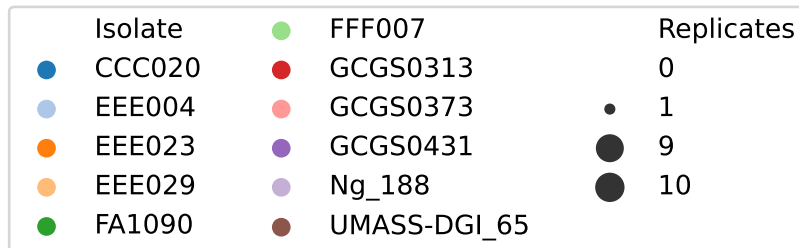

Supplement: S4 Fig — The number of changes in opa genes at 4 read coverage levels using Autocycler. For each isolate, the reads were randomly subsampled 10 times at each read coverage (replicates) and an assembly was created with the subsampled reads using Autocycler. The size of the point indicates the number of replicates. The changes at 50x coverage include one SNP in two separate genomes and one undetected opa, the change at 75x was multiple sequence differences in one genome, and the change at 125x coverage was one SNP in one genome. (PDF) [file ppat.1013962.s005.pdf]

# Complete genomes sequenced in this study

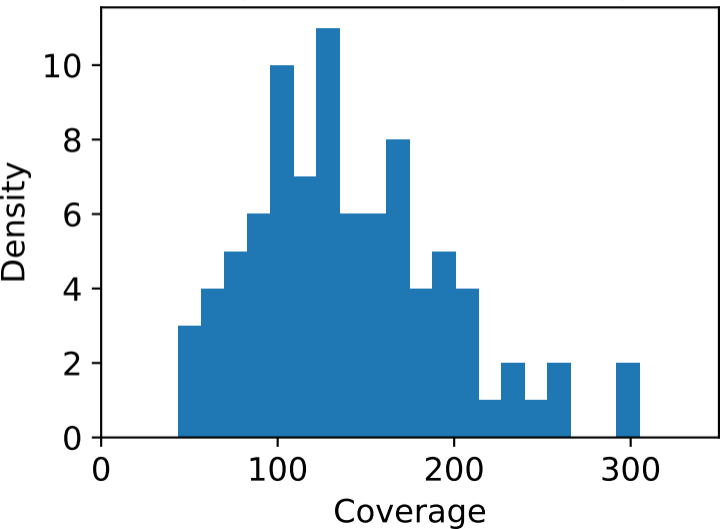

Supplement: S5 Fig — (PDF) [file ppat.1013962.s006.pdf]

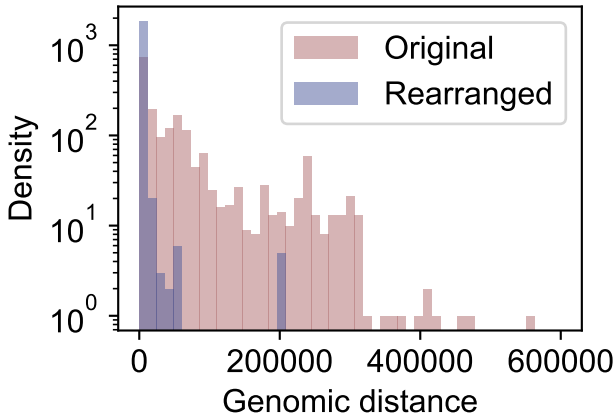

Supplement: S6 Fig — The distances are calculated as the distance between each opa in the reference genome FA1090 to the opa that is closest in genomic position in all other isolates that had 11 opa. (PDF) [file ppat.1013962.s007.pdf]

Reference genome (FA1090)

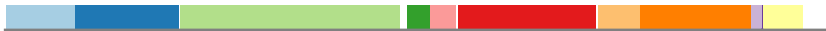

Query genome (DDD001)

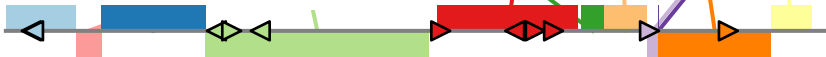

Query genome rearranged with respect to reference

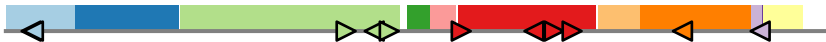

Supplement: S7 Fig — The shaded colored regions are the locally collinear blocks (LCBs). The LCBs that appear above the gray line are on the forward strand and those that appear below the gray line are on the reverse strand. A triangle pointing to the right indicates an opa gene on the forward strand and a triangle pointing to the left indicates an opa gene on the reverse strand. (PDF) [file ppat.1013962.s008.pdf]

a

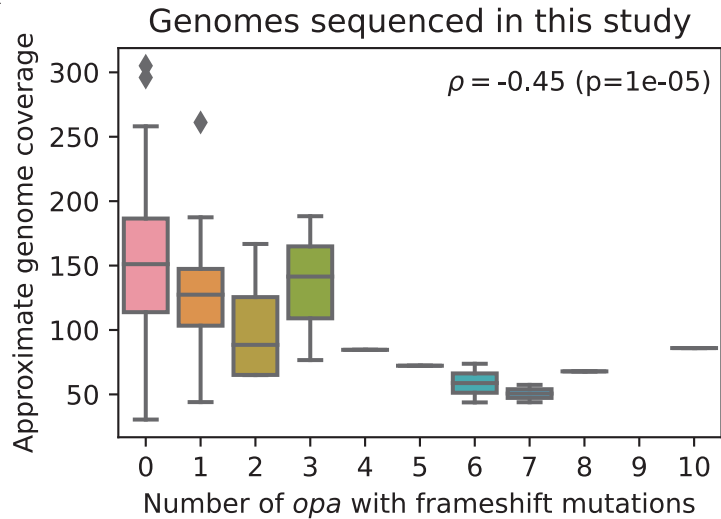

b

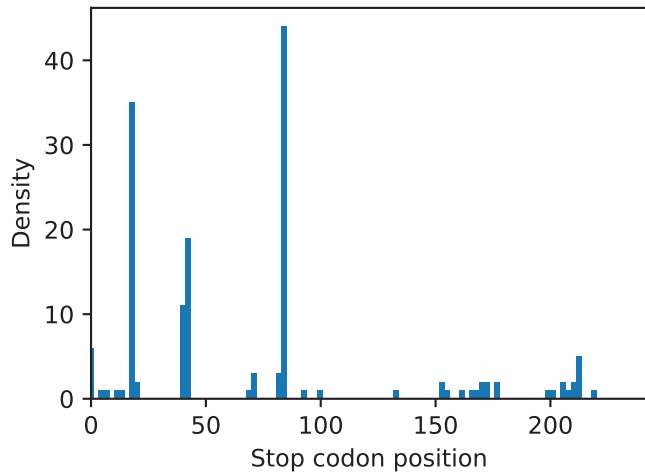

Supplement: S8 Fig — (a) The approximate genome sequencing coverage and number of opa in the genomes with frameshift mutations downstream of the coding repeats. (b) The locations of the stop codons in the opa genes with frameshift mutations downstream of the coding repeats. The maximum value of the x-axis is set at the average length of opa amino acid sequences. (PDF) [file ppat.1013962.s009.pdf]

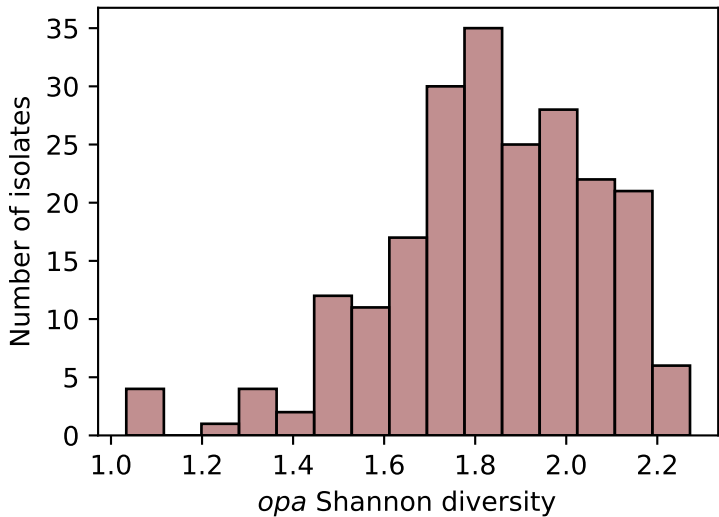

Supplement: S9 Fig — (PDF) [file ppat.1013962.s010.pdf]

Redundant *opa*

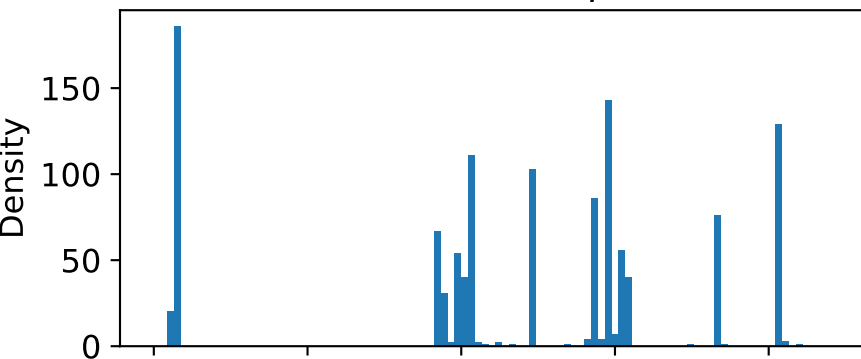

Unique *opa*

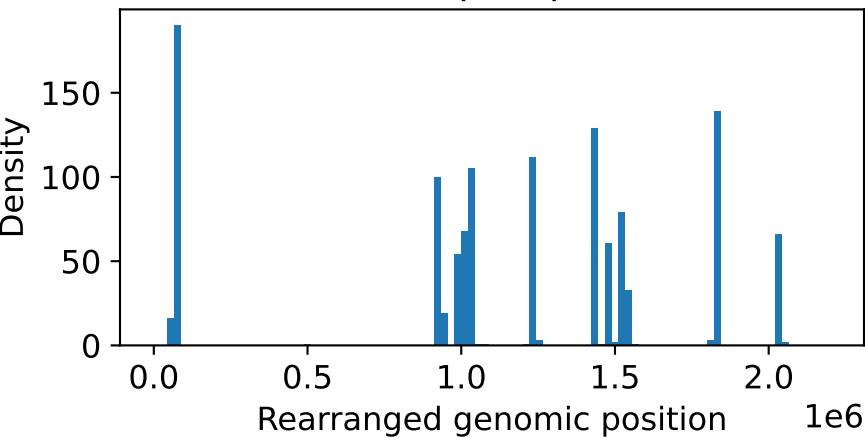

Supplement: S10 Fig — The rearranged genomic locations (using FA1090 as the reference genome) of the opa genes that are redundant (>95% amino acid identity within the same isolate) and opa genes that are unique (≤95% amino acid identity within the same isolate). The unique and redundant opa have a similar distribution of genomic locations. (PDF) [file ppat.1013962.s011.pdf]

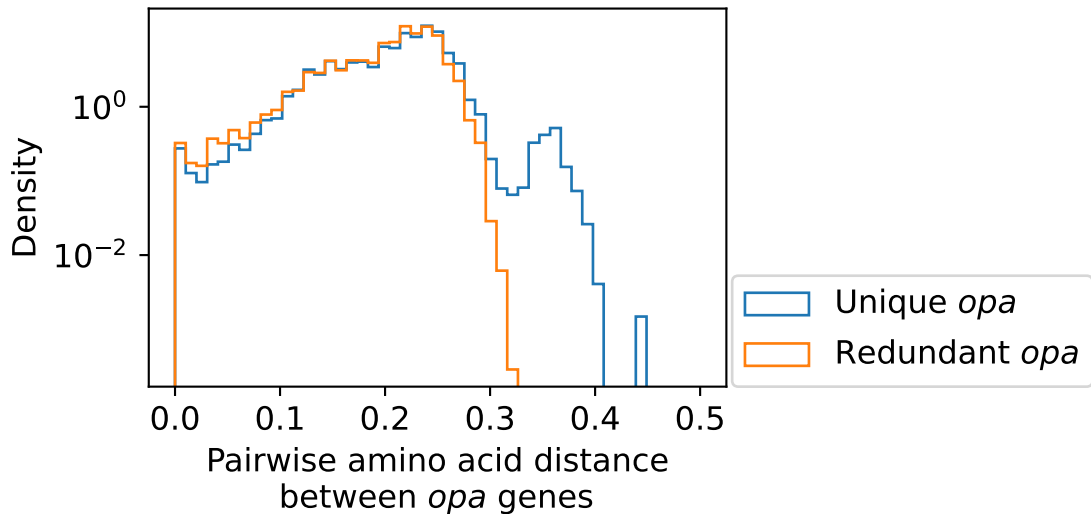

Supplement: S11 Fig — The distribution of pairwise amino acid distances between opa genes that are redundant (>95% amino acid identity within the same isolate) and opa genes that are unique (≤95% amino acid identity within the same isolate). The pairwise distance was calculated using only pairs of opa across isolates to not bias the calculation for redundant opa, which by definition have low pairwise distances when comparing within isolates. The distributions of pairwise distances are similar for unique and redundant opa, implying that the redundant opa do not have the same sequences across isolates. The peak at the right of the unique opa distribution is due to the 8 highly divergent opa sequences which do not have a redundant copy in the same isolate. (PDF) [file ppat.1013962.s012.pdf]

212/219 isolates have similar *opa* genes within the isolate

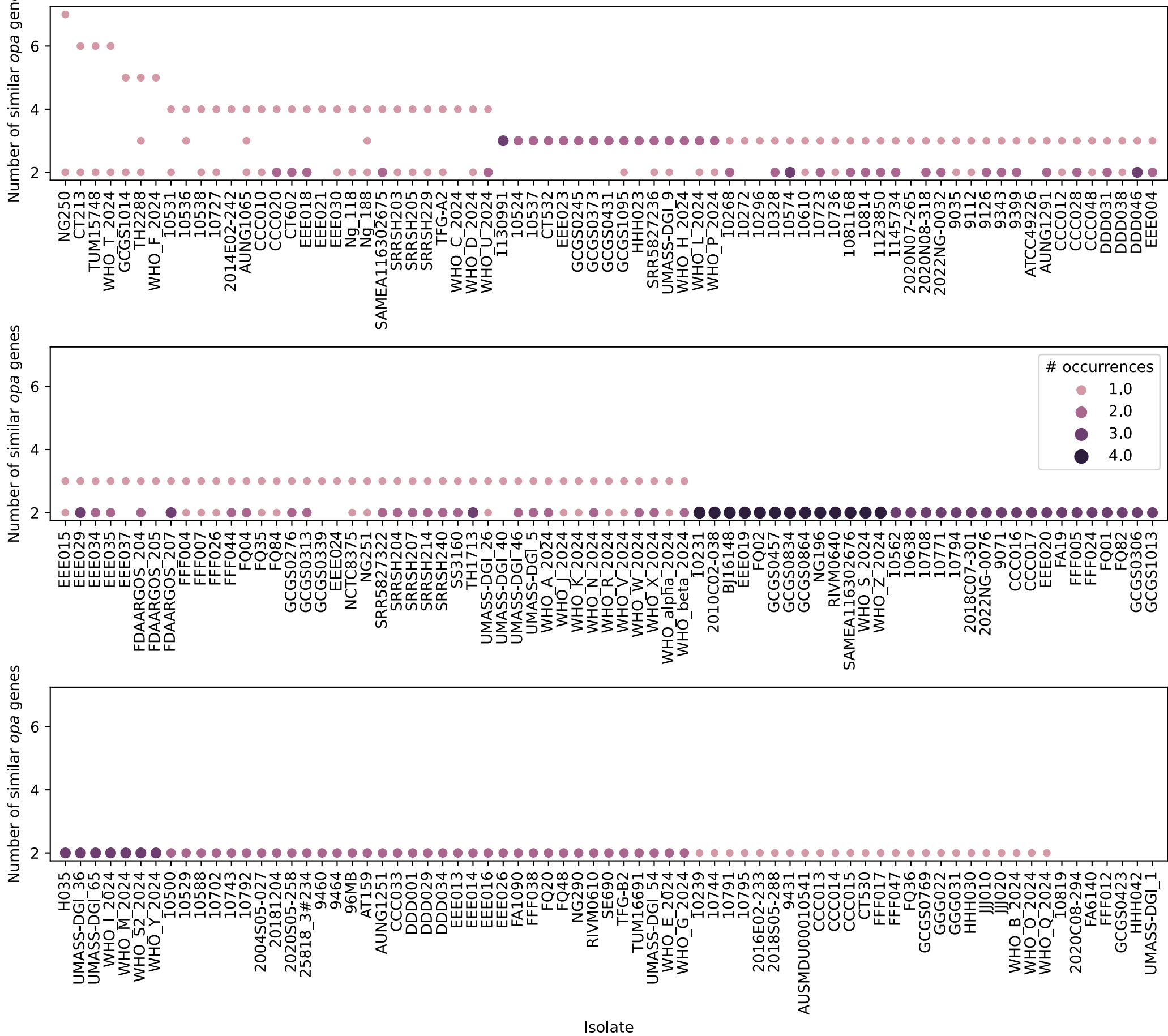

Supplement: S12 Fig — All isolates with complete genomes are shown on the x-axis. The points indicate groups of opa genes in the same isolate with >95% amino acid sequence identity. The y-axis shows the number of similar opa genes in each group. The size and color of the point indicate the number of distinct groups of each size in the genome. The x-axis is sorted first by the maximum number of similar opa genes in any group and then by the maximum number of groups. The plot is split into three rows for readability. (PDF) [file ppat.1013962.s013.pdf]

a

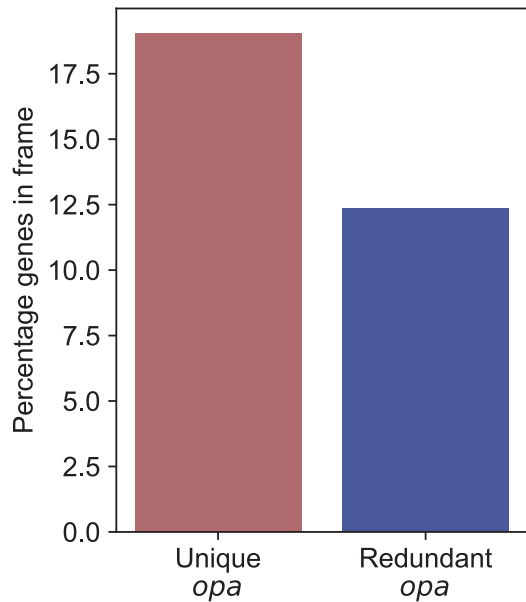

b

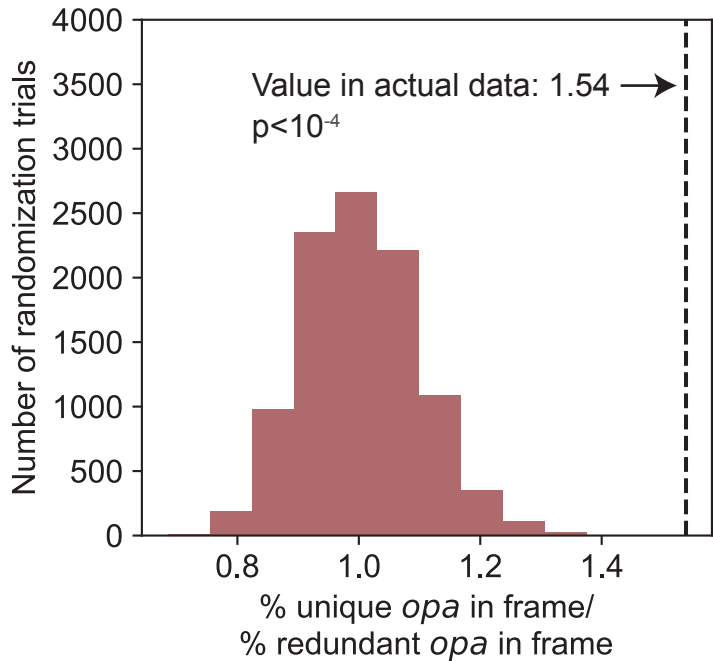

Supplement: S13 Fig — (a) The percentage of opa that are in frame for opa that are unique within an isolate (<95% amino acid similarity) or redundant within an isolate (≥95% amino acid similarity). (b) The ratio of the percentage of unique opa in frame to the percentage of redundant opa in frame for 104 randomizations of the data. The randomization procedure permuted which opa are labeled redundant, keeping the same total number of redundant opa across all isolates. Zero randomizations gave ratios as high as in the actual data (ratio of 1.54, indicated by the vertical dashed black line) yielding a p-value of less than 10-4. (PDF) [file ppat.1013962.s014.pdf]

Number of complete genomes

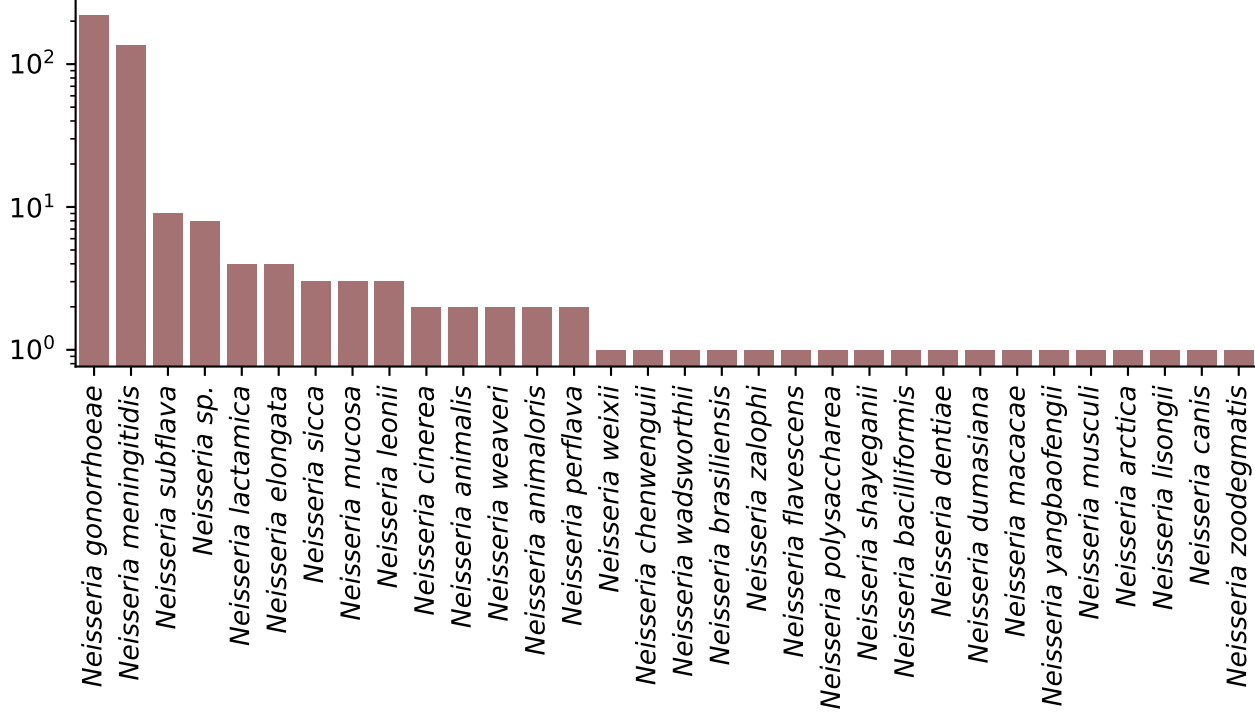

Supplement: S14 Fig — (PDF) [file ppat.1013962.s015.pdf]

a

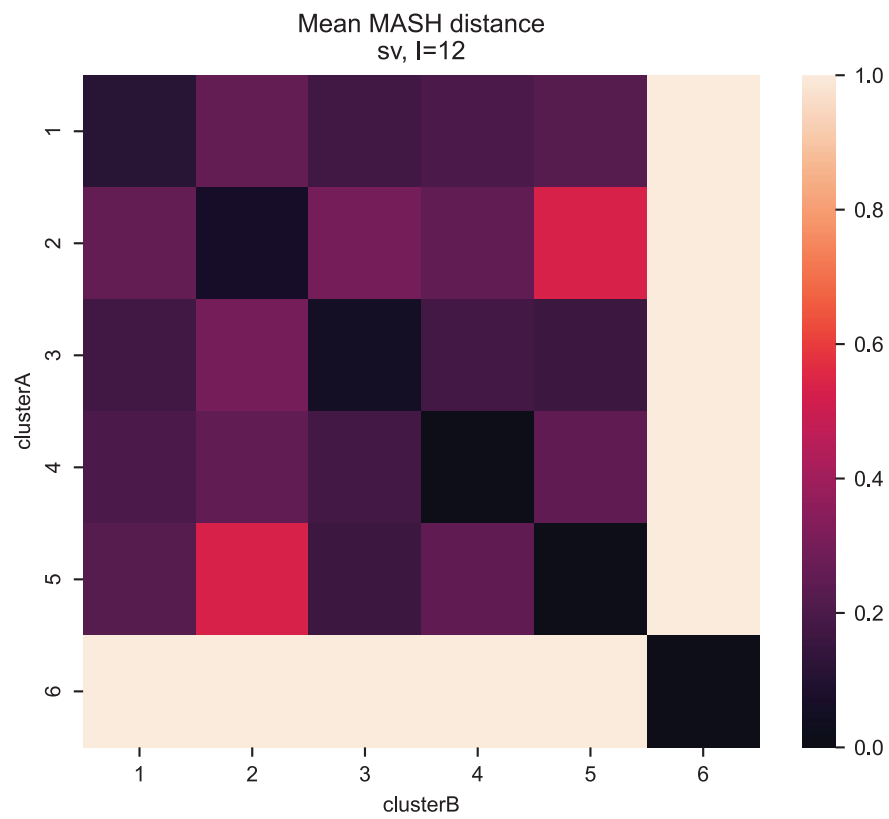

b

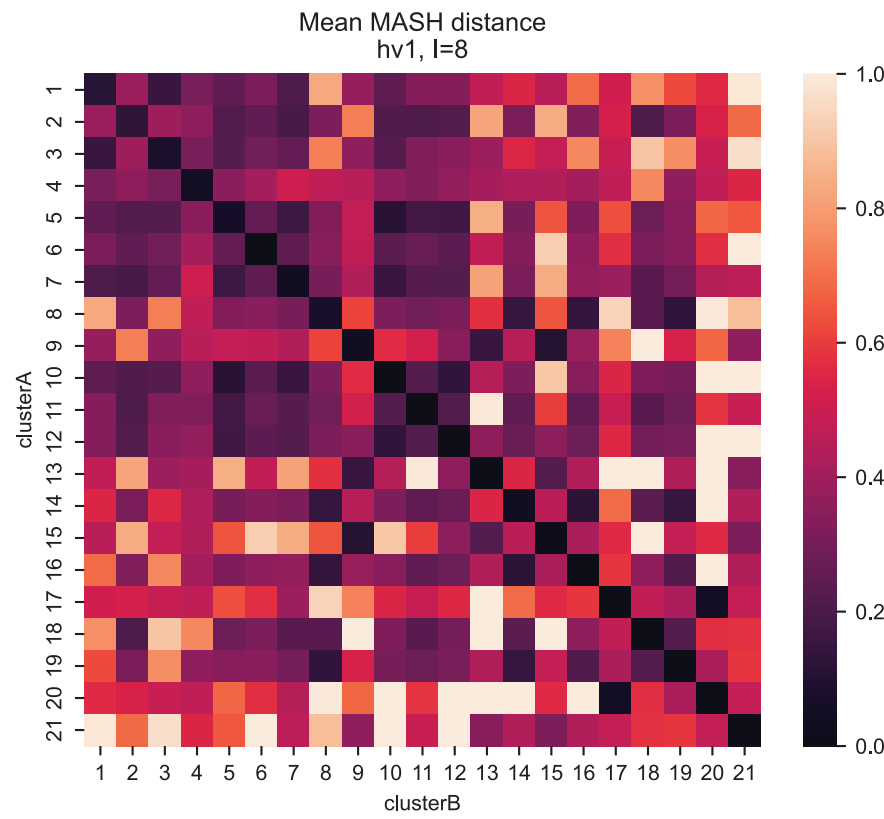

c

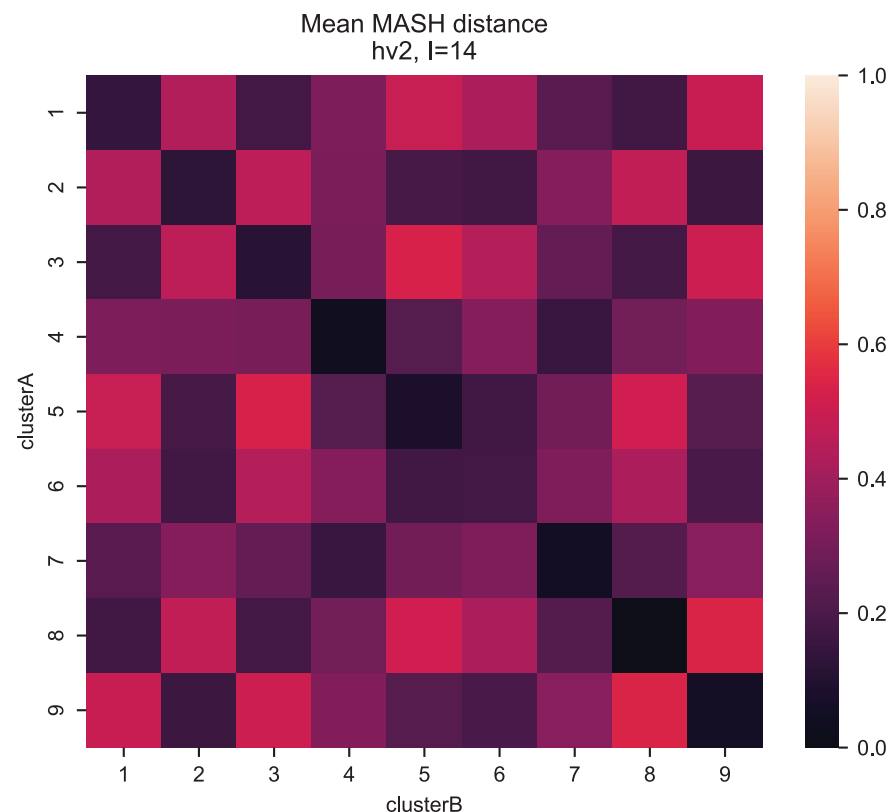

Supplement: S16 Fig — The mean nucleotide distance between pairs of sequences in the same and different clusters in the semivariable (a), hypervariable 1 (b), and hypervariable 2 (c) regions. (PDF) [file ppat.1013962.s017.pdf]

Number of sequences

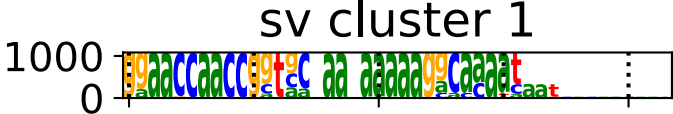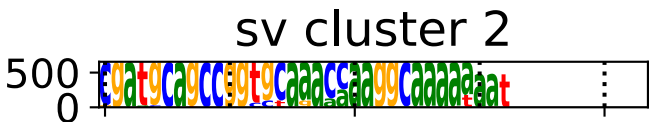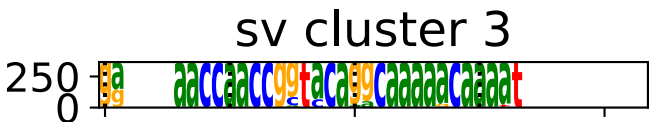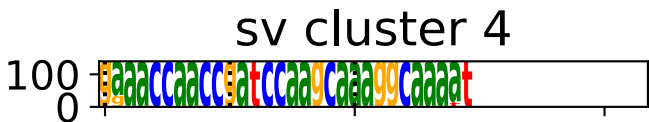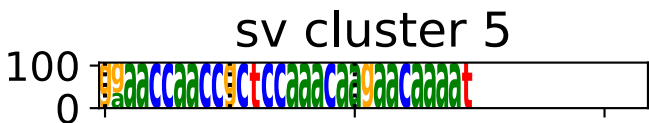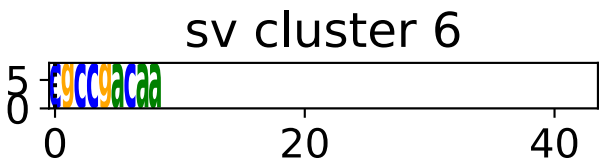

Supplement: S17 Fig — The nucleotide sequences were aligned using MAFFT in each cluster. The height of the nucleotides represents the number of sequences with each nucleotide. (PDF) [file ppat.1013962.s018.pdf]

Number of sequences

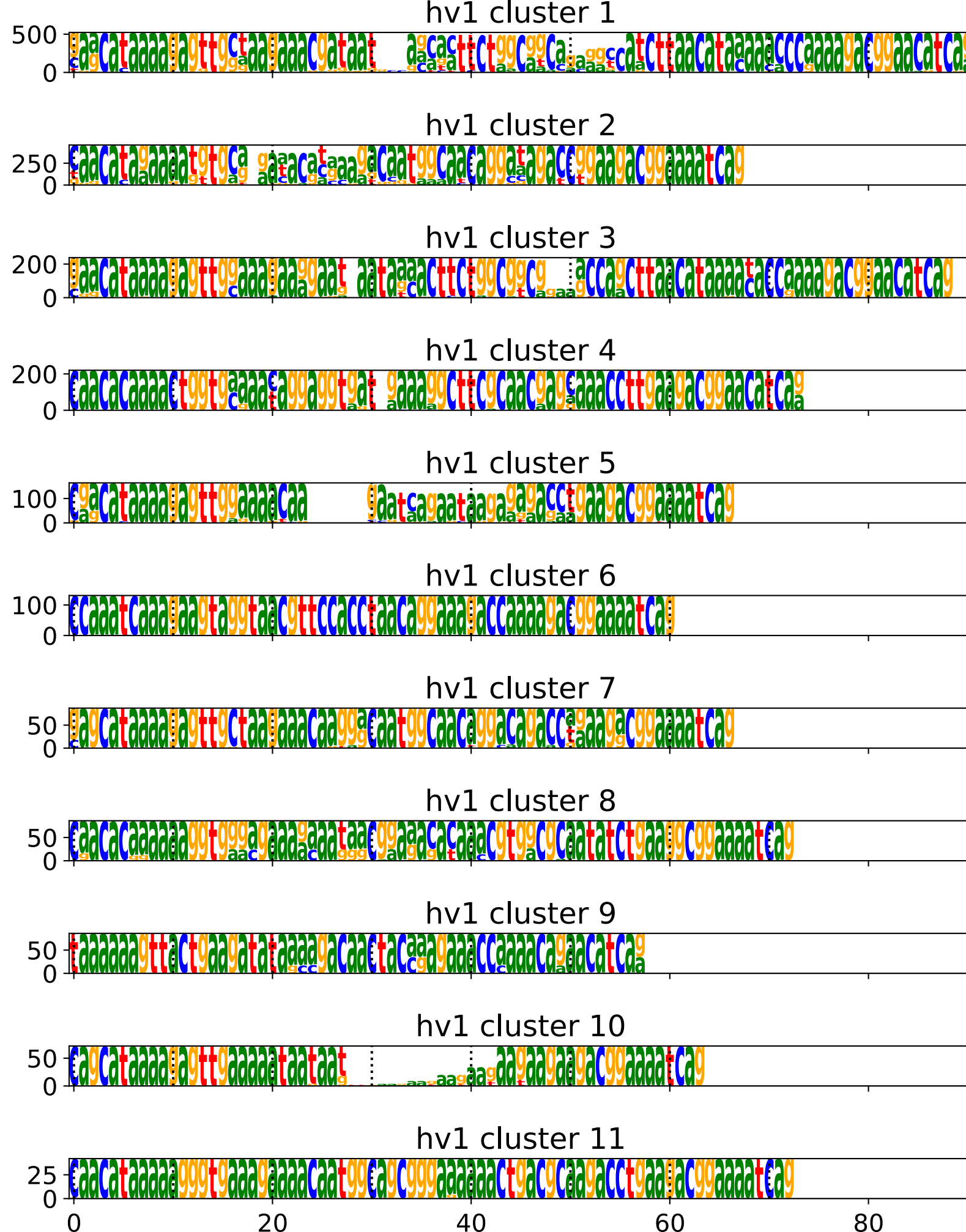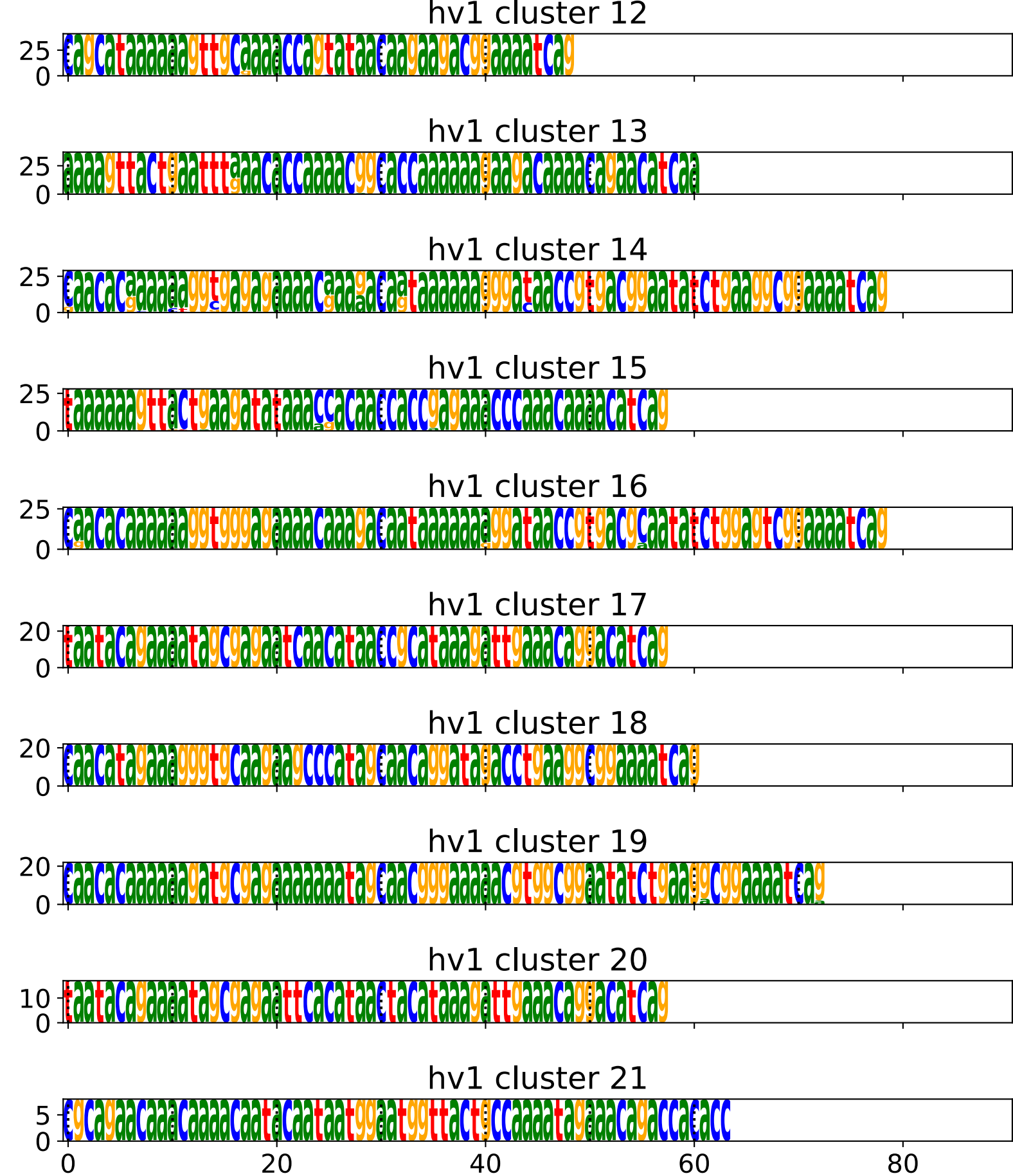

Supplement: S18 Fig — The nucleotide sequences were aligned using MAFFT in each cluster. The height of the nucleotides represents the number of sequences with each nucleotide. (PDF) [file ppat.1013962.s019.pdf]

Number of sequences

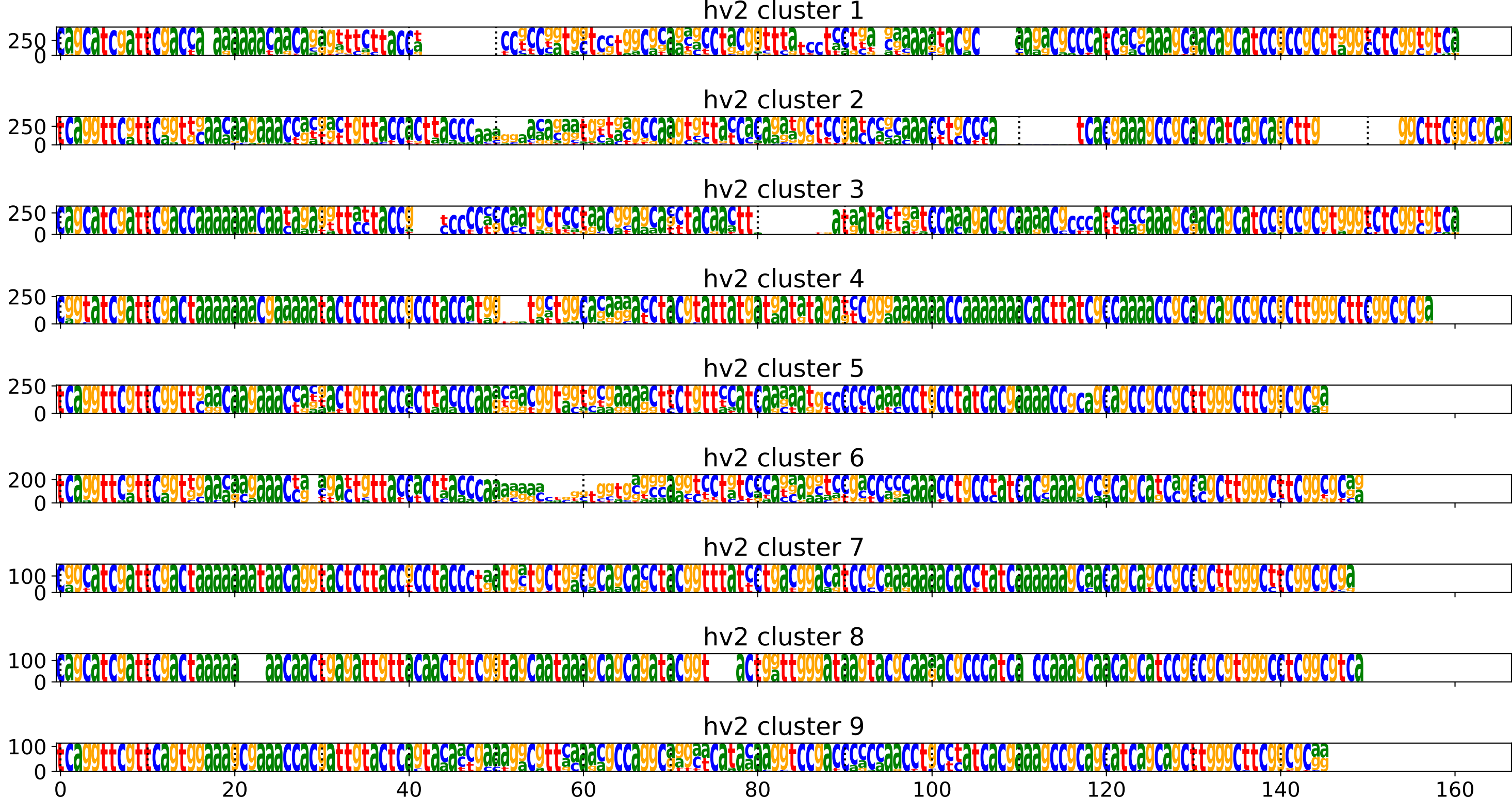

Supplement: S19 Fig — The nucleotide sequences were aligned using MAFFT in each cluster. The height of the nucleotides represents the number of sequences with each nucleotide. (PDF) [file ppat.1013962.s020.pdf]

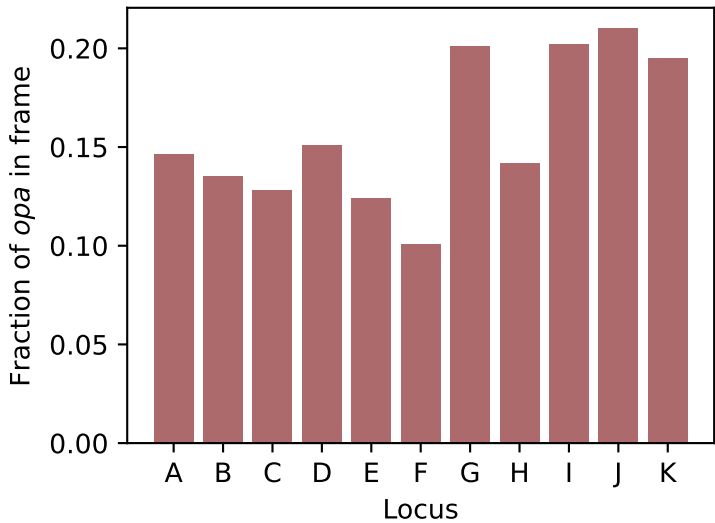

Supplement: S21 Fig — One-sided proportions Z-tests with a Bonferroni multiple hypothesis correction comparing the fraction of opa in frame at each locus (1) to the total fraction of opa in frame across all loci and (2) to the fraction of opaK alleles that are in frame are not significant. (PDF) [file ppat.1013962.s022.pdf]

a

Example of actual data

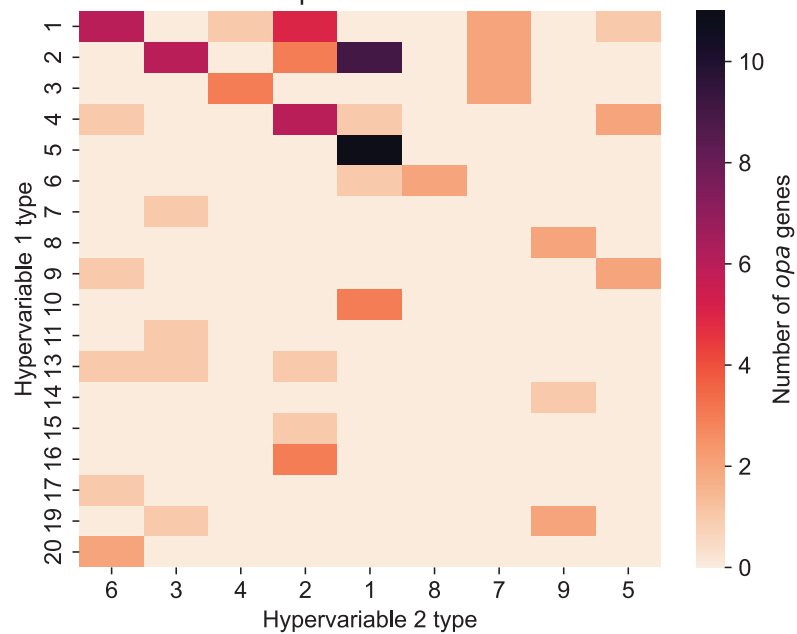

b

Example of randomized data

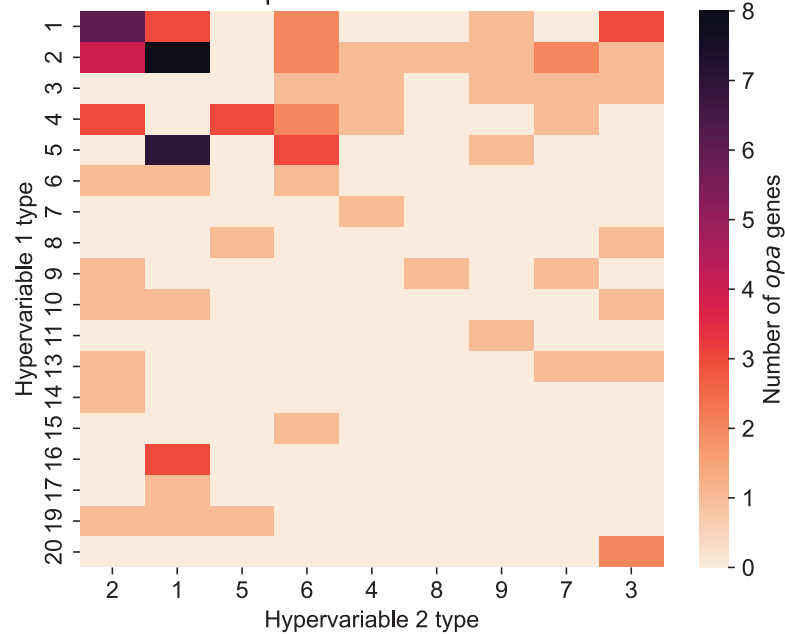

c

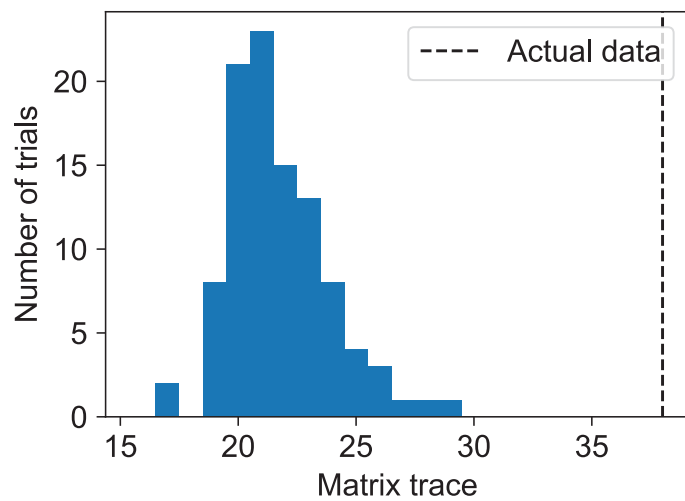

Supplement: S22 Fig — (a) The number of each combination of hypervariable 1 and hypervariable 2 types in opa alleles after accounting for isolate sampling and population structure. The columns of the matrix were rearranged to give the largest matrix trace (padding with columns of all zeros to make a square matrix). (b) The same data representation in (a) but after randomizing the assignment of hypervariable 2 types across the opa alleles. (c) The distribution of the maximum sum of the diagonal matrix elements (allowing for column rearrangements) in 100 randomizations of the hypervariable 2 type (blue histogram) compared to the actual data (black dashed line). (PDF) [file ppat.1013962.s023.pdf]
