# Supplementary material for: Diversity and evolution of a phase-variable multi-locus antigen in Neisseria gonorrhoeae
Source: PLoS Pathog. 2026 May 11;22(5):e1013962. doi: 10.1371/journal.ppat.1013962 (PMC13183285; doi:10.1371/journal.ppat.1013962)
Supplement: S15 Fig — (a) Schematic of the opa gene. The exact length and locations of the gene features varies across opa genes; depicted here is FA1090 opa1. CR: coding repeat, SV: semivariable region, HV1: hypervariable 1 region, HV2: hypervariable 2 region. (b) Summary of the approach to clustering variable region sequences. For each variable region (semivariable, hypervariable 1, and hypervariable 2), we calculated the k-mer distances between all sequences using MASH, setting k such that the probability of finding a random k-mer in each sequence is 0.01. We performed successive rounds of inflation (expansion and contraction) on the distance matrix, which amplifies high values of the matrix and suppresses low values of the matrix. We chose the lowest inflation parameter that gave a stable clustering. (c) The distribution of the cluster types for the sequences in each variable region. (PDF) [file ppat.1013962.s016.pdf]

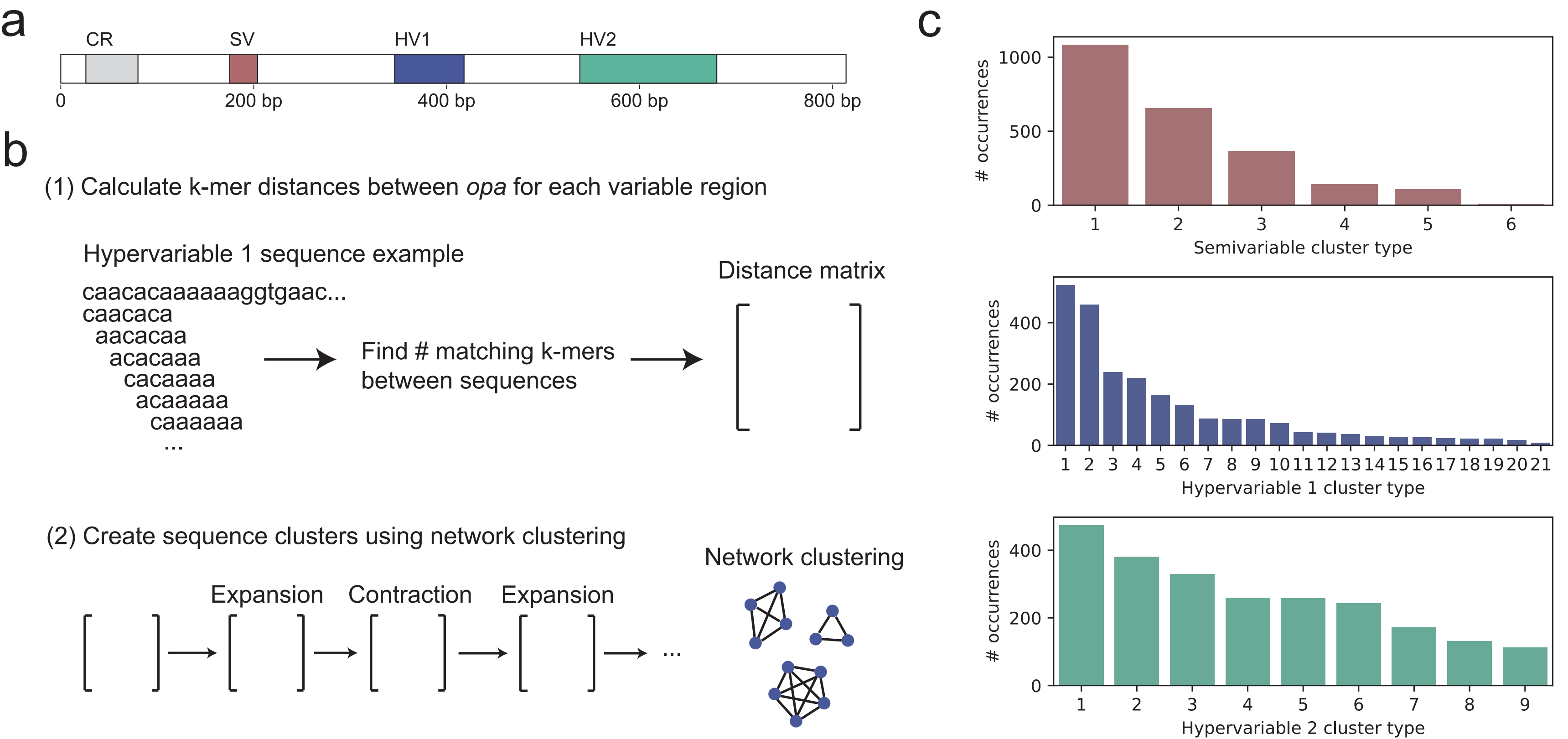
a
CR
SV
HV1
HV2
0
200 bp
400 bp
600 bp
800 bp
b
(1) Calculate k-mer distances between opa for each variable region
Hypervariable 1 sequence example
caacacaaaaaaggtgaac...
caacaca
aacacaa
acacaaa
cacaaaa
acaaaaa
caaaaaa
...
Find # matching k-mers between sequences
Distance matrix
(2) Create sequence clusters using network clustering
Expansion
Contraction
Expansion
...
Network clustering
c
occurrences
Semivariable cluster type
occurrences
Hypervariable 1 cluster type
occurrences
Hypervariable 2 cluster type
