# Supplementary material for: Diversity and evolution of a phase-variable multi-locus antigen in Neisseria gonorrhoeae
Source: PLoS Pathog. 2026 May 11;22(5):e1013962. doi: 10.1371/journal.ppat.1013962 (PMC13183285; doi:10.1371/journal.ppat.1013962)
Supplement: S20 Fig — The dated phylogeny of the subtree shown in Fig 6b annotated with the cluster types for the semivariable (a-b), hypervariable 1 (c-d), and hypervariable 2 (e-f) regions shown as colored circles for opaA (a, c, e) and opaK (b, d, f). The colors are comparable within each sequence region across loci (e.g., the same color scheme is used for the semivariable region of both opaA and opaK) but not between sequence regions. The colored circle annotations at the tips represent the cluster types of the isolates, and the colored circle annotations in the internal nodes represent the inferred ancestral state by PastML. The cluster types that are present in opaA and opaK are similar, but more closely related isolates have more similar cluster types in opaA compared to opaK. (PDF) [file ppat.1013962.s021.pdf]

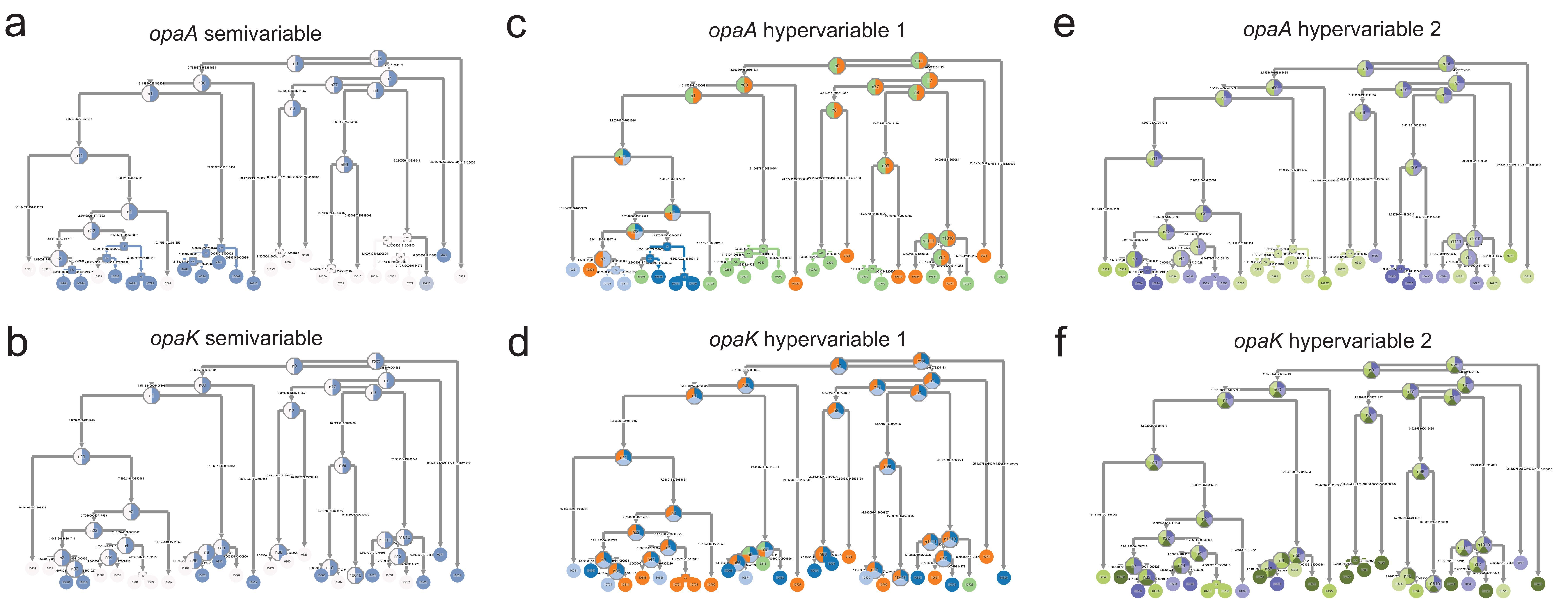
a
opaA semivariable
b
opaK semivariable
c
opaA hypervariable 1
d
opaK hypervariable 1
e
opaA hypervariable 2
f
opaK hypervariable 2
