# Supplementary material for: Diversity and evolution of a phase-variable multi-locus antigen in Neisseria gonorrhoeae
Source: PLoS Pathog. 2026 May 11;22(5):e1013962. doi: 10.1371/journal.ppat.1013962 (PMC13183285; doi:10.1371/journal.ppat.1013962)
Supplement: Attachment — Submitted filename: review.docx [file ppat.1013962.s032.docx]

This is a well-executed study that makes important contributions to our understanding of *Neisseria gonorrhoeae* *opa* diversity and evolution. The use of long-read sequencing to resolve multiple *opa* loci is methodologically sound, and the resulting dataset and analyses are comprehensive. The manuscript is generally well-written, and the findings are significant for vaccine development and immunological studies.

**Minor Comments**

I recommend publication, although the following minor issues should be addressed.

1. The algorithm for identifying *opa* genes (Supplementary Methods, lines 1079–1099) is complex. Consider adding a flowchart or decision tree in the main Methods section to improve accessibility.
2. The negative correlation between sequencing coverage and frameshift mutations suggests potential sequencing artefacts. The authors acknowledge but do not fully resolve this issue. The authors could provide a more detailed discussion of the threshold coverage at which frameshift mutations become reliable. Consider whether genomes below a certain coverage threshold should be excluded from downstream analyses, or clearly flag them as potentially unreliable.
3. The authors identify 98.8% of *opa* genes within locally collinear blocks with FA1090, but the remaining 1.2% (28 genes) are not accounted for in the locus assignment scheme. Could the authors clarify how these 28 genes are handled in downstream analyses. Provide a supplementary table listing these outliers and their genomic contexts.
4. The finding that fewer *opa* genes are in frame than expected by chance (mean 1.7 vs. expected 3.4, p < 0.001) is intriguing but the mechanistic explanation remains speculative. Expand the discussion of potential mechanisms (lines 639–644). The mention of promoter strength and progesterone-mediated selection is interesting but underdeveloped. Consider whether phase variation dynamics (i.e. the rate of frameshift/in-frame transitions) might differ between loci, which could explain the observed pattern.
5. The conclusion that "interspecies recombination does not appear to play a large role" (line 657) is based on the observation that only one *N. gonorrhoeae* *opa* allele clusters with commensal Neisseria *opa*. Acknowledge that this conclusion is based on a single observation and may not be definitive. Discuss the possibility that interspecies recombination events may be rare but historically important in shaping the *opa* repertoire. Consider whether the phylogenetic separation of *opa* between species could reflect ancient divergence rather than absence of recombination.

**Suggested wording changes:**

The manuscript is generally well-written, but a few sentences could be tightened.

6. Line 29: "Fewer *opa* genes were in frame, and thus inferred to be expressed, than expected due to chance." Consider: "Fewer *opa* genes were in frame (and thus inferred to be expressed) than expected by chance."
7. Line 72: "Studies of *opa* variation and evolution have focused on small numbers of isolates or closely related isolates." Consider: "Previous studies of *opa* variation and evolution have been limited to small numbers of isolates, often closely related."
