# Supplementary material for: Diversity and evolution of a phase-variable multi-locus antigen in Neisseria gonorrhoeae
Source: PLoS Pathog. 2026 May 11;22(5):e1013962. doi: 10.1371/journal.ppat.1013962 (PMC13183285; doi:10.1371/journal.ppat.1013962)
Supplement: Attachment — Submitted filename: response_to_reviewers.docx [file ppat.1013962.s034.docx]

**Part I - Summary**
Please use this section to discuss strengths/weaknesses of study, novelty/significance, general execution and scholarship.

We thank the reviewers for their helpful and insightful comments. We have addressed the points below and believe they have improved the content and readability of the manuscript. Please note that line numbers for the revised manuscript indicate those in the document with tracked changes.

Reviewer #1: The authors describe their work on various aspects of the opa genes in gonococci. Containing several opa genes that are subject to antigenic variation due to slip-strand mispairing evolutionary work has been hampered by sequencing technologies that heretofore were unsuitable to address the questions asked by the authors. Basic understanding of the evolution and variation of opa genes is critical for advancing knowledge regarding the contribution made by different Opa outer membrane proteins during infection and different forms of disease. To their credit the sequencing/bioinformatic approaches employed provide new insights. In general, the execution of the work was rigorous and the results are solid. While the scholarship is high I do have a few concerns that require attention.

1. The abstract refers to diverse clinical isolates but there is no description of them with respect to geographical location, gender or age of the infected, year of isolation or antibiotic resistance property. Such information should be provided to give the reader an idea as to the diverse nature of the isolates.

Thank you for the opportunity to clarify that we meant by “diverse clinical isolates”. We have updated the abstract and other places in the manuscript that refer to “diverse clinical isolates” to “phylogenetically diverse clinical isolates”, as shown in **Figure 1**. We have added information about the year of isolation and geographical location (continent) to the results section (lines 456-457). Where antibiotic resistance minimum inhibitory concentrations were available, we added them to the newly created **Table S6**. For all genomes, we also reported the antibiotic resistance alleles using the NG-STAR scheme in **Table S6** and we added the methods used to the Methods section. Unfortunately, most of the host gender and age information were not available.

*The isolates spanned 5 continents and the years 1962 to 2024 (****Figure S2****) and had a range of antibiotic resistance phenotypes and alleles (****Table S6****).*

**Figure S2: The distribution of continents, host genders, and years of isolation for the isolates that we sequenced from across the N. gonorrhoeae phylogeny (blue) and the publicly available complete genomes (orange).** The information on host gender was not included with NCBI metadata for the publicly available complete genomes. Abbreviations: Man (M), Woman (W), Not available (NA).

**Table S6:** **The NG-STAR types, associated antibiotic resistance markers, and available antibiotic minimum inhibitory concentrations for the genomes used in this study.** A value of "NA" in the NG-STAR type indicates that there was no NG-STAR type matching to the resistance profile. The 7 genes used for NG-STAR typing (penA, mtrR, porB, ponA, gyrA, parC, and 23S) and the alleles for each genome are shown. A value of "-" indicates that there was no matching allele in the NG-STAR database. The "AMR markers" columns report the AMR alleles. The "has_mics" column indicates whether minimum inhibitory concentrations (MICs) are available for each genome. Categorical resistance is denoted as "R" for resistant, "S" for susceptible, and "I" for intermediate.

2. The model shown in Fig. 7 is important. Unfortunately, there was seemingly no attempt to perform an in lab evolution experiment to test it. The absence of such a test should be incorporated into the limitations section within the Discussion.

Thank you for raising this point. We have added the following to the limitations section within the Discussion section (lines 939-940):

*Third, our study is observational and we have not performed experiments to test the model shown in Figure 7.*

3. While I agree that an Opa protein is likely to be only produced by a phase on gene it would be good to actually show this by western blotting. I suggest this because a promoter mutation could result in down-regulation of the cognate in-frame gene an this could influence biological events during infection.

We appreciate this suggestion and note that support for phase variation impacting translation is shown in ref. 23 (Stern et al., Cell, 1986).

4. The group recently published a manuscript in Nature Microbiology regarding fitness of gonococci during infection when the infecting strains differ in antibiotic resistance markers. If such recovered gonococci are available the manuscript would be strengthened if they followed the emergence of opa variants during experimental infection in the female mouse model that was used.

We thank the reviewer for this comment. We note that other work has looked at *opa*s during experimental infection in the female mouse model (Cole et al., Infection and Immunity, 2010), with the authors concluding that “Opa proteins promote persistence of *N. gonorrhoeae* in the female genital tract and that *opa* gene phase variation allows gonococci to evade or capitalize upon unidentified host factors of the mammalian reproductive cycle.” While this conclusion is from the mouse model of gonococcal colonization, it is consistent with the description of findings from human studies as described in the Introduction (c.f. paragraph 2 of the Introduction).

Reviewer #2: This is a well-executed study that makes important contributions to our understanding of Neisseria gonorrhoeae opa diversity and evolution. The use of long-read sequencing to resolve multiple opa loci is methodologically sound, and the resulting dataset and analyses are comprehensive. The manuscript is generally well-written, and the findings are significant for vaccine development and immunological studies.

We thank the reviewer for this supportive assessment.

Reviewer #3: This manuscript is a comprehensive investigation of the opacity-associated (Opa) protein gene family of Neisseria gonorrhoeae. Opa proteins are major adhesins and invasins for N. gonorrhoeae on human cells. Using publicly available genomes and genomes from strains sequenced by the authors, the authors develop a robust genomic and phylogenetic pipeline to identify putative opa genes, examine their synteny in aligned genomes, and predict their likelihood of expression based on the gene being in- or out-of-frame. This is an extensive, rich dataset that is a valuable addition to the Neisseria field. The findings, which are supported by the data, are interesting and insightful. I am very enthusiastic about this manuscript. My comments are intended to provide additional clarity and biological context for the general microbial pathogenesis audience of this study.

We thank the reviewer for this supportive assessment.

**Part II – Major Issues: Key Experiments Required for Acceptance**
Please use this section to detail the key new experiments or modifications of existing experiments that should be absolutely required to validate study conclusions.

Generally, there should be no more than 3 such required experiments or major modifications for a "Major Revision" recommendation. If more than 3 experiments are necessary to validate the study conclusions, then you are encouraged to recommend "Reject".

Reviewer #1: Points 3 and 4: the performance of these experiments is not critical but are only suggested to strengthen an already excellent paper.

We thank the reviewer and refer to our responses above to these points.

Reviewer #2: (No Response)

Reviewer #3: 1. Line 16, short title: Please do not use the term “antigenic variation” when describing Opa phase variation. In Neisseria, antigenic variation is shorthand for the recombination-based changes to the major pilin-encoding gene, so a reader could be confused by using antigenic variation to describe the Opa family. One suggestion is “(Landscape of) Opa variation in Neisseria gonorrhoeae”.

Thank you for raising this important point and for the helpful suggestion. We changed the short title to:

*Landscape of Opa variation in* Neisseria gonorrhoeae

Additionally, we updated lines 23-26 in the revised manuscript:

*Studies of Opa diversity and evolution have been limited by the inability of short-read sequencing to resolve the multiple copies of* opa *in each genome, preventing a comprehensive understanding of variation in gonococcal antigens for vaccine design and immunology studies.*

2. The study is inferring expression of Opa proteins from the putative translation of the DNA sequence in the sequenced genomes. However, Opa protein expression itself is not tested. Therefore, places that mention Opa expression should be reworded to “in frame”. This includes lines 50, 635, 637, 641.

We followed the reviewer’s suggestion, using “in frame” and related phrases instead of “Opa expression”, including:

Line 35: *Our findings reveal a continuously evolving* opa *repertoire that leads to diverse* opa *alleles even in closely related strains and indicate that there are likely unknown biological factors modulating* opa *reading frame.*

Line 60: *We found that Opa evolves rapidly to generate different versions of the protein in the same isolate, but very few of these protein versions are encoded by sequences that are in-frame.*

The subheading of the discussion in Line 736: *Quantitative assessment of* opa *diversity, reading frame, and evolution*

Line 738: *Are there patterns in their diversity and reading frame?*

Line 788: *Across the population, fewer opa were in frame than expected by chance.*

Line 798: *Nevertheless, our results suggest mechanisms other than chance alone in guiding the patterns of phase variation, for example, regulation of changes in repeat number or selection against isolates with many* opa *alleles in frame.*

Line 803: *Another potential mechanism is that there may be selection against in-frame opa in certain environments, for example the presence of progesterone [63–65] which has been shown to inhibit the growth of Opa positive isolates in-vitro.*

Line 954*: Taken together, our results support a model of opa variation where on short timescales phase variation modulates the translation of highly diverse opa alleles, typically with very few opa in frame at one time*

Caption of Figure 7 in line 973*: On short timescales, phase variation modulates the expression of highly diverse* opa *alleles within isolates, typically with very few* opa *being expressed at one time.*

3. For Figure 1, strains that were newly sequenced, how were the bacteria grown for isolating genomic DNA? Was there any phenotypic selection for Opa expression state? In vitro passaged bacteria may not be selected for their Opa expression, so the average number of phase-ON opa genes may be lower than observed in isolates directly obtained from human specimens with no or minimal passage. This point should also be addressed in lines 513-516, since publicly available genome sequences may have an over-representation of phase-OFF opa genes.

Thank you for this important point. We did not perform phenotypic selection for Opa expression state during bacteria growth for isolating genomic DNA. We have added this point to this discussion (line 793):

*We further note that the isolates we sequenced were not specifically selected for Opa expression state during culturing, so the average number of in frame opa genes may be lower than observed in isolates directly obtained from human specimens with no or minimal passaging. Similarly, publicly available genomes may have an over representation of out of frame opa genes due to unknown culturing conditions.*

We also updated the limitations section of the discussion (line 935):

*Second, gonococcal passaging during culturing may have changed the phase of opa genes and specifically could have decreased the number of in frame* opa *genes due to Opa expression not being selected for in laboratory culture.*

4. Discussion: With the many subheadings, this section is disjointed, and in some places reiterates the Results without additional context. The Discussion could be reorganized to combine some of the small sections into paragraphs, each with conclusions and future directions associated with them.

We have reorganized the Discussion so that the section “Quantitative assessment of *opa* diversity, frame, and evolution” is now organized into two main subsections: “Number, phase, and locations of *opa* genes” and “*opa* diversity and evolution*”* and the smaller sections have been combined into paragraphs within these subsections.

a. Line 618, can you speculate the mechanism for the other loss events – are there repeat sequences in or near the opa genes where the losses are seen?

We updated line 749 which now reads:

*We were unable to identify the mechanism for the other loss events, which may have resulted from larger deletions in surrounding regions of the genome, since we could not find remnants of these opa genes by homology search methods.*

b. Line 628-633: are the duplicated opa genes always found in the same locations in each genome? Is it always the same pairs of opa genes that are duplicated?

Thank you for raising these interesting questions. We performed additional analyses on the duplicated *opa* genes and observed that they are not always found in the same genomic location and they do not have similar sequences to one another across isolates. The following has been added to the revised manuscript (lines 598):

*The redundant opa genes were not always found in the same location in the genome and did not have similar sequences to one another (****Figure S10****,* ***Figure S11****).*

**Figure S10**: **Redundant opa genes are not always found in the same locations across isolates.** The rearranged genomic locations (using FA1090 as the reference genome) of the opa genes that are redundant (>95% amino acid identity within the same isolate) and opa genes that are unique (≤95% amino acid identity within the same isolate). The unique and redundant opa have a similar distribution of genomic locations.

**Figure S11:** **Redundant opa do not have the same sequences across isolates.** The distribution of pairwise amino acid distances between opa genes that are redundant (>95% amino acid identity within the same isolate) and opa genes that are unique (≤95% amino acid identity within the same isolate). The pairwise distance was calculated using only pairs of opa across isolates to not bias the calculation for redundant opa, which by definition have low pairwise distances when comparing within isolates. The distributions of pairwise distances are similar for unique and redundant opa, implying that the redundant opa do not have the same sequences across isolates. The peak at the right of the unique opa distribution is due to the 8 highly divergent opa sequences which do not have a redundant copy in the same isolate.

c. Line 635 section, and also sentence on line 694-695: Missing is the concept of phenotypic redundancy: that Opa proteins lack sequence homology but retain receptor binding characteristics. In this context, it is important to include that receptor binding property cannot be predicted from opa sequence.

Thank you for raising this important point. We have added to the text (line 788 in revised manuscript):

*Furthermore, near-identical* opa *genes in the same isolate tended to be out of frame more often than unique* opa *genes, suggesting selection against expression of redundant* opa*; we note, however, that the receptor binding characteristics of Opa proteins cannot be predicted from* opa *sequence alone and there may be additional phenotypic redundancy, i.e., Opa proteins with low sequence homology may also share receptor binding characteristics.*

We updated line 798 in revised manuscript (line 636 in original submission), which previously read:

*Our results suggest mechanisms other than chance alone in guiding the patterns of phase variation, for example, regulation of changes in repeat number or selection against isolates with many opa alleles in frame.*

And now reads:

*Nevertheless, our results suggest mechanisms other than chance alone in guiding the patterns of phase variation, for example, regulation of changes in repeat number, selection against isolates with many opa alleles in frame, or selection for Opa proteins with specific receptor binding characteristics.*

We also added to the discussion (line 944 in revised manuscript):

*However, this classification scheme may miss some phenotypic redundancy, as Opa proteins that lack high sequence homology can retain receptor binding characteristics. Currently, the receptor binding properties of Opa proteins cannot be predicted from opa sequence.*

d. Line 652: the terminology implies that N. gonorrhoeae can regulate the on/off expression of opa genes (“turned on in subsequent reinfections”). Opa genes undergo phase variation at random, and then there is selection for or against individual bacteria in a population. This diversity helps the population as a whole to maintain infectivity and survival.

We changed the sentence in line 837 in the revised manuscript (line 648 in original submission) which previously read (bolded part has been changed):

*We speculate that if there is immunological memory to the opa that are expressed, then* ***different opa are turned on in subsequent reinfections****, an idea that is also supported by evidence of variation in opa expression over the course of single infections in human challenge studies.*

And now reads:

*We speculate that if there is immunological memory to the opa that are expressed, then* ***there will be selection in subsequent reinfections for isolates that have different opa turned on****, an idea that is also supported by evidence of variation in opa expression over the course of single infections in human challenge studies.*

e. Section starting on line 679, add citations for studies describing the non-random association of particular hypervariable loop 1 and 2 sequences (PMID: 1809845, PMID: 1694004). Also add that swapping hypervariable loops does not lead to predictable interpretations of receptor binding (reference 67). And line 693, add references #8 and 11 along with #15.

We have added references PMID 1809845 and PMID 1694004 to the section starting in line 908 in the revised manuscript (line 679 in the original manuscript) which previously read:

*We showed that this phenomenon also occurs in N. gonorrhoeae.*

And now reads:

*We showed that this phenomenon also occurs in N. gonorrhoeae, which is consistent with studies showing non-random associations of particular hypervariable region 1 and 2 sequences [69,70].*

We added the following sentence to the end of this section:

*Previous work has shown that swapping the hypervariable region sequences between opa genes does not lead to predictable changes in receptor binding* [72]*, underscoring the importance of functional coordination between these regions and providing a plausible mechanism for the selection of specific combinations.*

We have added references #8 and 11 along with #15 in line 942 in the revised manuscript (line 945 in the original submission).

f. “Genomic rearrangements shuffle opa position” is a less major conclusion than others in this manuscript, and I would not lead off the discussion with this point.

We moved this part to the end of the subsection in the Discussion, “Number, phase, and locations of *opa* genes”.

**Part III – Minor Issues: Editorial and Data Presentation Modifications**
Please use this section for editorial suggestions as well as relatively minor modifications of existing data that would enhance clarity.

Reviewer #1: Please see points 1 & 2 above.

Reviewer #2: Minor Comments

I recommend publication, although the following minor issues should be addressed.

1. The algorithm for identifying opa genes (Supplementary Methods, lines 1079–1099) is complex. Consider adding a flowchart or decision tree in the main Methods section to improve accessibility.

Thank you for this helpful suggestion. We added the following flowchart as a supplementary figure (**Figure S1**) and referenced it in the Methods section (line 173 in the revised manuscript). We didn’t add it as a figure in the main methods because we felt that the current Figure 1 is more appropriate as the first main text figure.

**Figure S1: Flow chart summarizing algorithm for identify opa genes.** Note that a mismatch refers to a substitution, insertion, or deletion.

2. The negative correlation between sequencing coverage and frameshift mutations suggests potential sequencing artefacts. The authors acknowledge but do not fully resolve this issue. The authors could provide a more detailed discussion of the threshold coverage at which frameshift mutations become reliable. Consider whether genomes below a certain coverage threshold should be excluded from downstream analyses, or clearly flag them as potentially unreliable.

In our simulation tests of the effect of sequencing coverage on the reconstructed *opa* sequence (Figure S4), we found that down to 50x, the *opa* sequences were still almost identical to the *opa* sequences from genomes with 125x coverage. The differences that were observed were SNPs, which would not cause frameshift mutations, and one example each of a missing *opa* (which would also not be a frameshift mutation) and multiple sequence differences in a single *opa* (the only example that would lead to frameshift mutations). Thus, our data suggest that coverages down to 50x would generally be reliable. This is consistent with what is recommended by the developers of Autocycler (Wick et al., 2025). Furthermore, in our preliminary testing of 2 genomes (Figure S3), we found that down to 21x, the *opa* sequences from the Autocycler assemblies were still identical to the *opa* sequences from genomes with >140x coverage. Thus coverages down to 21x could also be reliable.

The complete genomes that we sequenced have coverage between 30x and 305x with an average coverage of 140x. While there are submitter-reported coverages for the publicly available genomes on NCBI, we found that they were unreliable as some coverages were identical for different genomes from the same submitter, suggesting that they may have been placeholders and were not calculated directly from the data. As a result, we could not easily obtain accurate coverages for the publicly available data.

We added the following to the revised manuscript (lines 472):

*The genomes that we sequenced had coverages between 44x and 305x with an average coverage of 141x, with 84/87 (96.6%) above the threshold of 50x that we tested and all genomes above the threshold of 21x (****Figure S5****).*

**Figure S5: The distribution of read coverages for the genomes that we sequenced in this study**.

We were not able to fully resolve the issue for the observed frameshift mutations because while our simulation tests suggest that frameshift mutations due to sequencing error at the coverage levels in these genomes should be rare, we nonetheless have not directly tested it for these sequences.

We added the following to the discussion section of the revised manuscript (lines 755):

*Our simulation studies indicated that genome coverages down to 50x are reliable and those down to 21x could also be reliable. The genomes that we sequenced have coverage above this range. We were unable to conclusively determine the coverages of the publicly available complete genomes. While our simulation tests suggest that frameshift mutations due to sequencing error at the coverage levels for the complete genomes that we sequenced should be rare, we could not fully resolve the issue for the observed frameshift mutations.*

3. The authors identify 98.8% of opa genes within locally collinear blocks with FA1090, but the remaining 1.2% (28 genes) are not accounted for in the locus assignment scheme. Could the authors clarify how these 28 genes are handled in downstream analyses. Provide a supplementary table listing these outliers and their genomic contexts.

The 28 genes that did not fall within locally collinear blocks with FA1090 were kept in their original positions. In the original manuscript lines 179-180 (revised manuscript lines 235), we stated:

*Regions of the query genome that did not fall into any locally collinear block with the reference genome were not altered.*

We added **Table S7** listing the *opa* genes that did not fall into any locally collinear block with the reference genome and referenced it in the revised manuscript in lines 541. We also realized that the previously summary statistic (2331/2359 *opa* genes within locally collinear blocks) had included the FA1090 *opa* genes and have removed them from the denominator to more accurately represent this result (2331/2348 *opa* genes within locally collinear blocks):

*We found 2,331/2,348 (99.3%)* *opa genes within locally collinear alignment blocks with the FA1090 genome (note that the 11 FA1090 opa genes have been removed from this calculation). The opa genes that did not fall into any locally collinear alignment blocks with the FA1090 genome are listed in* ***Table S7.***

***Table S7: opa genes that did not fall into any locally collinear alignment blocks with the FA1090 genome.*** *The opa ID is a name that’s given based on the location that the opa genes occur in the query genome.*

| **Strain** | ***opa* ID** | **Start position** | **Stop position** | **Strand** |
| --- | --- | --- | --- | --- |
| 10272 | 10272_opa_6 | 1497887 | 1498706 | -1 |
| 10574 | 10574_opa_6 | 1471128 | 1471921 | -1 |
| 10610 | 10610_opa_10 | 2102791 | 2103614 | -1 |
| 1123850 | 1123850_opa_3 | 494891 | 495718 | -1 |
| 9399 | 9399_opa_6 | 1497876 | 1498699 | -1 |
| AT159 | AT159_opa_1 | 70615 | 71421 | -1 |
| AUNG1065 | AUNG1065_opa_1 | 69325 | 70199 | -1 |
| CCC028 | CCC028_opa_10 | 1737074 | 1737899 | 1 |
| EEE034 | EEE034_opa_8 | 1525041 | 1525877 | 1 |
| GCGS0245 | GCGS0245_opa_8 | 1348523 | 1349315 | 1 |
| NG196 | NG196_opa_6 | 1124580 | 1125415 | 1 |
| NG290 | NG290_opa_6 | 1125670 | 1126493 | 1 |
| TUM15748 | TUM15748_opa_1 | 69329 | 70153 | -1 |
| UMASS-DGI_26 | UMASS-DGI_26_opa_2 | 74272 | 75057 | -1 |
| WHO_C_2024 | WHO_C_2024_opa_8 | 1469376 | 1470145 | 1 |
| WHO_L_2024 | WHO_L_2024_opa_11 | 2052838 | 2053632 | -1 |
| WHO_T_2024 | WHO_T_2024_opa_6 | 1163549 | 1164352 | 1 |

The genes that did not fall within the loci definitions were labeled as “Other”. We added the following to the results section of the revised manuscript (lines 548):

opa *genes that did not fall within the definitions of the loci were given the label “Other” (****Figure 2b-c, Table S8****).*

We added **Table S8** with the list of *opa* genes that were given the label “Other”.

**Table S8: *opa* genes that did not fall within the definitions of the loci and were given the locus label “Other”.** The *opa* ID is a name that’s given based on the location that the *opa* genes occur in the query genome.

| **Strain** | ***opa* ID** | **Start position** | **Stop position** | **Strand** | **Start position mapped to FA1090 genome** | **Strand mapped to FA1090 genome** |
| --- | --- | --- | --- | --- | --- | --- |
| 10610 | 10610_opa_10 | 2102791 | 2103614 | -1 | 2102791 | -1 |
| 10743 | 10743_opa_8 | 1534646 | 1535430 | 1 | 75671 | -1 |
| 1123850 | 1123850_opa_3 | 494891 | 495718 | -1 | 494891 | -1 |
| AT159 | AT159_opa_9 | 1790846 | 1791697 | 1 | 75496 | -1 |
| AUNG1065 | AUNG1065_opa_7 | 1515225 | 1516099 | 1 | 75450 | -1 |
| CCC028 | CCC028_opa_10 | 1737074 | 1737899 | 1 | 1737074 | 1 |
| DDD029 | DDD029_opa_9 | 1453001 | 1453807 | 1 | 75704 | -1 |
| DDD034 | DDD034_opa_9 | 1453062 | 1453873 | 1 | 75703 | -1 |
| DDD038 | DDD038_opa_9 | 1452168 | 1452974 | 1 | 75703 | -1 |
| GCGS0245 | GCGS0245_opa_8 | 1348523 | 1349315 | 1 | 1348523 | 1 |
| NG196 | NG196_opa_6 | 1124580 | 1125415 | 1 | 1124580 | 1 |
| NG290 | NG290_opa_6 | 1125670 | 1126493 | 1 | 1125670 | 1 |
| TUM15748 | TUM15748_opa_7 | 1557757 | 1558566 | 1 | 75551 | -1 |
| UMASS-DGI_26 | UMASS-DGI_26_opa_2 | 74272 | 75057 | -1 | 74272 | -1 |
| WHO_T_2024 | WHO_T_2024_opa_6 | 1163549 | 1164352 | 1 | 1163549 | 1 |

Note that the *opa* with locus label “Other” can include the genes that did not fall within locally collinear blocks, but not necessarily, and can also include other genes, as it is only defined based on the rearranged genomic positions of the genes. The genes with the locus “Other” were excluded from downstream analysis, in particular the analysis of rate of *opa* evolution (**Figure 6**) because they are not necessarily in the same locus.

4. The finding that fewer opa genes are in frame than expected by chance (mean 1.7 vs. expected 3.4, p < 0.001) is intriguing but the mechanistic explanation remains speculative. Expand the discussion of potential mechanisms (lines 639–644). The mention of promoter strength and progesterone-mediated selection is interesting but underdeveloped. Consider whether phase variation dynamics (i.e. the rate of frameshift/in-frame transitions) might differ between loci, which could explain the observed pattern.

We expanded on the discussion of the potential mechanisms in the discussion. It previously read in the original manuscript (line 638):

*While these mechanisms are yet unknown, previous work suggested that promoter strength may play a role in regulating opa phase [59] and there may be selection against opa expression in certain environments, for example the presence of progesterone [60–62].*

And now reads in the revised manuscript (line 801, bolded parts are new):

*While these mechanisms are yet unknown, previous work suggested that promoter strength may play a role in regulating opa phase [59]****, and this could potentially regulate changes in repeat number. Another potential mechanism is that*** *there may be selection against in-frame opa in certain environments, for example the presence of progesterone [60–62],* ***which has been shown to inhibit the growth of Opa positive isolates in vitro.***

While the reviewer raises an interesting point about whether the phase variation dynamics might differ between loci, we unfortunately don’t have data on the dynamics as we only have a snapshot in time.

5. The conclusion that "interspecies recombination does not appear to play a large role" (line 657) is based on the observation that only one N. gonorrhoeae opa allele clusters with commensal Neisseria opa. Acknowledge that this conclusion is based on a single observation and may not be definitive. Discuss the possibility that interspecies recombination events may be rare but historically important in shaping the opa repertoire. Consider whether the phylogenetic separation of opa between species could reflect ancient divergence rather than absence of recombination.

Thank you for these helpful suggestions. In the original manuscript (lines 656-659), we acknowledged that sequencing more genomes may lead to more observations of interspecies recombination:

*While sequencing more complete genomes may lead to more observations of interspecies mosaicism, the separate clustering of N. gonorrhoeae and N. meningitidis opa in our dataset and in Malorny et al.* [9] *suggests phylogenetic separation of opa sequences between species.*

We added the following to the discussion (lines 892 in revised manuscript) about the possibility of historical interspecies recombination in shaping the *opa* repertoire:

*It is possible that interspecies recombination was historically important in shaping the opa repertoire and that the phylogenetic separation of opa between species may reflect ancient divergence.*

Suggested wording changes:

The manuscript is generally well-written, but a few sentences could be tightened.

Thank you for these helpful suggestions. These changes have been implemented in the revised manuscript.

6. Line 29: "Fewer opa genes were in frame, and thus inferred to be expressed, than expected due to chance." Consider: "Fewer opa genes were in frame (and thus inferred to be expressed) than expected by chance."

7. Line 72: "Studies of opa variation and evolution have focused on small numbers of isolates or closely related isolates." Consider: "Previous studies of opa variation and evolution have been limited to small numbers of isolates, often closely related."

Reviewer #3: 1. Terminology on Line 21, Line 63: The full name for Opa is opacity-associated protein. Opa proteins are a family of related proteins, so would reword to (line 21) “One of the most abundant and diverse antigens are members of the opacity-associated (Opa) family, surface proteins that mediate gonococcal attachment…” and (line 63) “A major family of surface-exposed proteins that undergo this variation are the opacity-associated (Opa) proteins”.

Thank you for raising this helpful point. We have reworded the full name for Opa to opacity-associated protein in the locations mentioned. Additionally, line 54 in the author summary have been updated to:

*One of the most abundant surface-exposed proteins are members of the opacity-associated (Opa) family, which help N. gonorrhoeae bind to host cells upon colonization.*

2. Line 26, Line 360, Line 375, elsewhere: Please define what is “diverse” about the clinical isolates.

Revised manuscript lines 27, 60, 448, 468, 1343, 1541, 1627 have been updated to say “phylogenetically diverse” isolates or clinical isolates.

3. Line 64: Replace “host receptors” with “human receptors” to emphasize these effects are human-specific. Also add that Opa proteins mediate non-opsonic phagocytosis by human neutrophils (cite PMID: 8971715, PMID: 8962144, PMID: 9218786).

Lines 23, 77, 913, 941 have been updated from “host receptors” to “human receptors”.

We added this sentence to lines 78-79 in the revised manuscript:

*Opa proteins also mediate non-opsonic phagocytosis by human neutrophils [19–21].*

4. Line 68: Add that the pentanucleotide repeats are in the signal sequence-encoding region of the opa gene.

Line 90 in the revised manuscript has been updated to read (bold highlights addition):

*A series of pentanucleotide repeats in the* ***signal sequence-encoding region at the*** *5’ end of the coding sequence…*

5. Line 77: add the connection between Opa phase variation and opa gene promoter strength, reference 59.

The connection between Opa phase variation and *opa* gene promoter strength has been added to lines 100-101 in the revised manuscript:

*There is evidence of correlation between promoter strength and the rate of opa phase variation [26].*

6. Lines 163-165: please clarify this wording.

Lines 163-165 in the original manuscript previously read:

*We looked at gene clusters that included the opa genes identified by our method to assess for additional genes in those clusters not identified by our method.*

We updated this in the revised manuscript (lines 212-214) to read:

*We looked at genes that clustered with the opa genes identified by our method to determine if there were any additional genes in these clusters not identified by our method.*

7. Line 168: Seems like it would be more accurate to call the opa genes missing the start or stop codon pseudogenes, not “truncated,” which implies a premature stop codon or an internal deletion.

Lines 216-217 (lines 168-169 in original manuscript) have been updated from “truncated *opa*” to “*opa* pseudogenes”. The following locations in the revised manuscript have also been updated: lines 228, 483, 487, 495, 496, 524, 526, 651, and 749. In some of these locations (lines 483 and 749), we also clarified to say “*opa* pseudogenes that were missing the beginning or end of the gene” since there are other ways to form a pseudogene.

8. Line 389: describe what makes these 8 opa genes divergent. Do they have the pentanucleotide repeats and conserved parts of the opa sequence?

These 8 *opa* genes had the pentanucleotide repeats but the conserved and semivariable parts of the *opa* sequence were divergent. We added this to line 485 in the revised manuscript:

*These genes had the pentanucleotide repeats but were divergent in the conserved and semivariable parts of the opa sequence.*

Interestingly, these are the same 8 *opa* genes that clustered more with *opas* from other *Neisseria* species in our analysis of interspecies recombination. We added this to line 665 in the revised manuscript:

*We note that these 8 opa genes were divergent in the conserved parts of the sequence compared to other N. gonorrhoeae opa and were the same 8 opa genes missed by BLAST in our original opa search.*

9. Line 416: how many is a “handful of times”?

We kept this vague because to really know the number of times *opa* was lost, we would need to do a more rigorous ancestral state reconstruction analysis. However, we updated the manuscript to now say (lines 518-519 in revised manuscript):

*Based on the genomes in this study, we observed opa losses up to around 15 times across the phylogeny (Figure 2a).*

This is based on the fact that we see 15 independent clusters of genomes in the phylogeny that are nested within genomes that have higher numbers of *opa* genes. However, there could have been fewer loss events, if, for example, there were two closely related clusters with fewer *opa* genes that resulted from one loss event and one gain event. There could have also been more loss events, if, for example, there were two neighboring isolates on the phylogeny that each lost an *opa* in independent events.

10. Figure on line 485 should be deleted.

We did not see a figure in line 485 of our manuscript, but we have checked that there were no spurious figures in the manuscript.

**References**

Cole, Jessica G., Nanette B. Fulcher, and Ann E. Jerse. 2010. “Opacity Proteins Increase Neisseria Gonorrhoeae Fitness in the Female Genital Tract Due to a Factor under Ovarian Control.” *Infection and Immunity* 78 (4): 1629–41.

Stern, A., M. Brown, P. Nickel, and T. F. Meyer. 1986. “Opacity Genes in Neisseria Gonorrhoeae: Control of Phase and Antigenic Variation.” *Cell* 47 (1): 61–71.

Wick, Ryan R., Benjamin P. Howden, and Timothy P. Stinear. 2025. “Autocycler: Long-Read Consensus Assembly for Bacterial Genomes.” *Bioinformatics (Oxford, England)* 41 (9): btaf474.
